# Colloidal Perovskite Nanocrystals for Blue‐Light‐Emitting Diodes and Displays

**DOI:** 10.1002/advs.202409736

**Published:** 2025-03-09

**Authors:** Md Aftabuzzaman, Yongju Hong, Sangyeon Jeong, Ratiani Levan, Seung Jin Lee, Dong Hoon Choi, Kwangyeol Lee

**Affiliations:** ^1^ Department of Chemistry and Research Institute for Natural Sciences Korea University Seoul 02841 Republic of Korea

**Keywords:** colloidal perovskite nanocrystals, perovskite light‐emitting diode, quantum confinements, quantum dots and display

## Abstract

The evolution of display technology toward ultrahigh resolution, high color purity, and cost‐effectiveness has generated interest in metal halide perovskites, particularly colloidal perovskite nanocrystals (PeNCs). PeNCs exhibit narrow emission spectra, high photoluminescence quantum yields, and wide color gamuts, rendering them promising candidates for next‐generation displays. Despite significant advancements in perovskite light‐emitting diode (PeLED) technology, challenges remain regarding the efficiencies of PeNC‐based blue LEDs. Addressing these challenges, including both inherent and external instabilities of PeNCs and operational instabilities of the devices, is important as they collectively impede the broader acceptance and utilization of PeNCs. Herein, a comprehensive overview of the syntheses of dimension‐ and composition‐controlled blue colloidal PeNCs and critical factors influencing the performances of colloidal PeNC‐based blue LEDs is provided. Moreover, the advancements of colloidal PeNC‐based blue LEDs and challenges associated with the application of these LEDs are explored, and the potentials of these LEDs for application in next‐generation displays are emphasized. This review highlights the path forward for the future development of PeNC‐based blue LEDs.

## Graphical abstract

This review provides a comprehensive overview of the synthesis of colloidal perovskite nanocrystals and explores the key factors that influence the performance of PeNC‐based blue LEDs. It also discusses the challenges and recent advancements in applying these nanocrystals in blue LEDs.

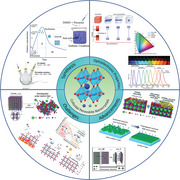

## 1 Introduction

**Figure 1:**
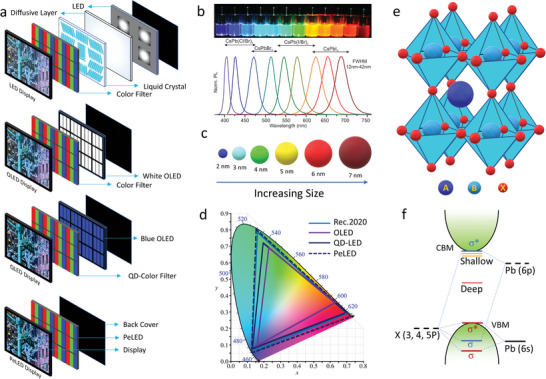
a) Evolution of display technology from liquid crystal‐based LEDs to next‐generation PeLEDs. b) Composition‐dependent entire color spectrum of colloidal PeNCs. Reproduced with permission.^[^ ^10^ ^]^ Copyright 2015, American Chemical Society. c) Color dependency of QDs as a function of size. d) The CIE chromaticity diagram and the color gamut coverage of OLEDs, conventional QD LEDs, and PeLEDs. The data points for this figure are obtained from the ref. [29, 30, 31, 32, 33, 34, 35, 36] e) Crystal structure of perovskite, and f) their bandgap origination. Adapted with permission.^[^ ^37^ ^]^

Over the past 20 years, display technology has undergone significant transformations (**Figure** **1**a). Currently, the display market is dominated by organic light‐emitting diodes (OLEDs), demonstrating high efficiencies and long lifetimes. However, OLEDs exhibit low color purities due to broad full width at half maximum (FWHM) values (> 45 nm) of their emission spectra and cover only 65% of the color gamut Rec. 2020, which is insufficient for high‐color‐purity displays.^[^ ^1^, ^2^, ^3^ ^]^ CdSe/S and InP quantum dots (QDs), emerging as viable contenders for OLEDs, demonstrate relatively narrow FWHM values of ≈20–40 nm and wide color gamuts when used as a backlight source for liquid‐crystal displays (≈110% of National Television Standards Committee (NTSC) on the Commission Internationale de l'Éclairage (CIE) chromaticity diagram).^[^ ^1^, ^4^ ^]^ However, CdSe/S and InP QDs also fail to meet the requirement of 85% color gamut of Rec. 2020 for application in high‐color‐purity displays.^[^ ^3^, ^5^, ^6^ ^]^ Use of vacuum evaporation in OLED displays and complex synthesis of both organic fluorophores and defect‐free, highly pure conventional QDs contribute to the high costs of OLEDs and conventional QD LEDs, which necessitate the search for new, more affordable display materials.^[^ ^1^, ^3^ ^]^

Next‐generation displays should offer ultrahigh resolutions, high color purities, and low costs. Metal halide perovskites (MHPs) have emerged as a new class of luminescent materials, demonstrating high color purities with narrow FWHM values of the emission spectrum (12–25 nm), high photoluminescence quantum yields (PLQYs) (≈100%), and wide color gamuts (140% NTSC on the CIE chromaticity diagram), which meet the standard of Rec.2020.^[^ ^7^, ^8^, ^9^, ^10^, ^11^ ^]^ Moreover, the band gaps and colors of MHPs depend on both the compositions and sizes of MHPs (Figure 1b–d). MHPs consist of an ABX_3_ structure, where A is an organic ammonium (e.g., methylammonium (MA; CH_3_NH_3_^+^) and formamidinium (FA; CH(NH_2_)_2_^+^)) or an alkali‐metal cation (e.g., Cs^+^), B is a transition metal cation (e.g., Pb^2+^, Au^2+^, Sn^2+^, and Mn^2+^), and X is a halide anion (including I^−^, Br^−^, and Cl^−^). MHPs exhibit very high defect tolerances with easily tunable band gaps, which can be adjusted by simply changing the X components (Figure 1e).^[^ ^8^, ^12^ ^]^ Moreover, MHPs can be synthesized in various forms (e.g., single crystals, polycrystalline bulk films, and colloidal nanocrystals) due to their compatibilities with various solution processes.^[^ ^2^, ^13^ ^]^ Bulk MHPs demonstrate comparatively low exciton binding energies of 26–150 meV and longer carrier diffusion lengths (1 µm) than those of organic materials (10 nm).^[^ ^14^, ^15^, ^16^ ^]^ Therefore, the exciton can travel to the defect site and EML interface, where deep‐level traps cause non‐radiative recombination. In contrast, colloidal perovskite nanocrystals (PeNCs) confine charge carriers to small regions and prevent the dissociation of excitons into free charge carriers.^[^ ^17^, ^18^ ^]^ Unlike the cases of CdSe/S and InP QDs, most defects in colloidal PeNCs are shallow and can be passivated by ligand engineering (Figure 1f).^[^ ^19^ ^]^ Furthermore, PeNCs allow core–shell‐type structure engineering with high efficiency and desirable stability.^[^ ^20^, ^21^, ^22^ ^]^ **Table** **1** presents a comparison of the physical and optoelectronic properties of PeNCs, organic fluorophores, and CdSe/S and InP QDs.

**Table 1:** Physical and optoelectronic properties of PeNCs, organic fluorophores, and conventional QDs.

Parameters	Organic fluorophores	Conventional QDs (CdSe/S, InP)	Perovskites
Typical λ_PL_ range (nm)	420‐650^[^ ^38^, ^39^, ^40^, ^41^, ^42^ ^]^	450‐650^[^ ^1^, ^43^, ^44^, ^45^ ^]^	410‐850^[^ ^10^, ^12^, ^46^, ^47^, ^48^ ^]^
PL FWHM (nm)	> 45^[^ ^39^, ^49^ ^]^	20‐40^[^ ^43^ ^]^	12–25
PLQY (%)	≈90^[^ ^39^, ^49^ ^]^	> 90^[^ ^43^, ^50^ ^]^	≈100^[^ ^9^ ^]^
Color Gamut (%) (NTSC standard)	≈90^[^ ^39^, ^51^ ^]^	≈110^[^ ^1^, ^4^ ^]^	≈140^[^ ^3^, ^10^ ^]^
Molar absorption coefficient (×10^6^ M^−1^ cm^−1^)	< 1^[^ ^52^, ^53^, ^54^ ^]^	≈1–10^[^ ^55^ ^]^	≈10–50^[^ ^56^, ^57^, ^58^ ^]^
Stability	Medium	High	Medium
^a^RoHS regulation (ppm)	No issue	Cd (100)	1000
Cost ($/g)	10–50	5–15	≈2
Rec.2020 agreement (%)	≥ 60	90	100

^a)^ Restriction of Hazardous Substances.

Because of these underlying advantages of PeNCs, many efforts have been made to improve the efficiencies of perovskite LEDs (PeLEDs). The green and red PeLEDs have achieved impressive external quantum efficiencies (EQEs) of 28.9%^[^ ^23^ ^]^ (at 540 nm) and 28.7%^[^ ^24^ ^]^ (at 638 nm), respectively, within a short development period. Although the highest EQE for blue‐emitting PeLEDs has reached 26.4% (at 480 nm),^[^ ^25^ ^]^ achieving high efficiency for blue‐emitting PeLEDs remains challenging. The difficulties arise from synthesizing stable blue perovskite materials, managing high defect density and deep‐level defects, as well as ensuring efficient charge injection and transport.^[^ ^26^, ^27^, ^28^ ^]^ For full‐color wide‐gamut PeLEDs, improving the EQEs of blue LEDs is important. Despite their current lagging performances, PeLEDs hold significant promise in the future LED horizon because the compositions and morphologies of colloidal PeNCs can be facilely tuned, which considerably affects their photophysical properties. Furthermore, colloidal PeNCs can be assembled into 2D films in LEDs, and their morphology‐dependent assembly behaviors are closely linked to the device's performance. Therefore, in this review, we focus on the exciting behaviors of colloidal PeNC‐based blue LEDs and discuss the key factors influencing the efficiencies and stabilities of PeNC‐based blue LEDs to guide further performance improvement and practical applications of these LEDs.

## 2 Device Physics and Working Principle of PeLED

**Figure 2:**
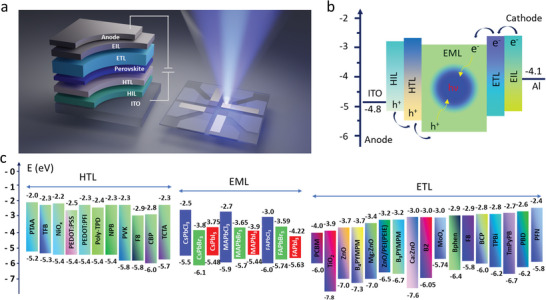
a) Illustration of the PeLED device structure along with its emission during operation. Adapted with permission.^[^ ^66^ ^]^ b) The operating mechanism of the PeLED. c) The energy levels of typical HTL, ETL, and EML used in PeLED.^[^ ^25^, ^28^, ^61^, ^62^, ^67^, ^68^, ^69^, ^70^, ^71^, ^72^, ^73^, ^74^, ^75^, ^76^, ^77^, ^78^, ^79^, ^80^ ^]^

A typical PeLED structure, illustrated in **Figure** **2**a, consists of multiple layers including indium tin oxide (ITO) anode, hole injection layer (HIL), hole transport layer (HTL), emissive layer (EML), electron transport layer (ETL), electron‐injection layer (EIL), and cathode. When applying forward bias on the electrodes, holes, and electrons are first injected into HIL and EIL, respectively. The anode is responsible for injecting holes into the EML. Directly on top of the anode is the HIL, which facilitates efficient hole transfer from the anode into the EML. A commonly used HIL in PeLEDs is poly(3, 4‐ethylenedioxythiophene)‐poly(styrenesulfonate) (PEDOT:PSS).^[^ ^59^, ^60^ ^]^ To further enhance charge transport and achieve a better balance between the PEDOT:PSS and the EML, an HTL, such as poly[*N, N*″‐bis(4‐butylphenyl)‐*N, N*″‐bis(phenyl)‐benzidine] (Poly‐TPD), and poly (9‐vinylcarbazole) (PVK) is added.^[^ ^61^, ^62^ ^]^ Both the HIL and HTL are typically conductive polymers.

On the cathode side, electrons are injected into the EML. The ETL plays a key role in transferring electrons from the cathode to the EML. A commonly used ETL is 1, 3, 5‐tris(1‐phenyl‐1H‐benzimidazol‐2‐yl)benzene (TPBi).^[^ ^63^, ^64^ ^]^ Between the ETL and the cathode, an EIL, such as lithium fluoride (LiF),^[^ ^65^ ^]^ is typically inserted to enhance electron injection by tuning the work function of the cathode. The cathode itself is usually made of a low‐work‐function metal like aluminum (Al), which aids in efficient electron injection. At the core of the structure is the EML. The injected electrons from the cathode and holes from the anode recombine in the EML, producing light. Figure 2b illustrates the working principle of the PeLED.

The conversion efficiency of the PeLED is defined as the ratio of the number of photons generated to the number of electrons passing through the LED called EQE. It can be expressed as

(1) $$\begin{array}{ccc} \mathrm{EQE}\left(\%\right) & = & \mathrm{Number}\;\mathrm{of}\;\mathrm{photons}\;\mathrm{emitted}\\ & & /\mathrm{Number}\;\mathrm{of}\;\mathrm{charges}\;\mathrm{injected}=\eta_{\mathrm{inj}}\times \eta_{\mathrm{rad}}\times \eta_{\mathrm{out}} \end{array}$$

where *η*_inj_, *η*_rad_, and *η*_out_ are the efficiency of charge injection, radiative recombination, and light output, respectively. *η*_inj_ refers to the effectiveness with which charge carriers are injected from the electrodes into the active layer of the device, where they recombine to emit light. This can be achieved by optimizing the energy band alignment between the electrodes and charge‐transport layers, ensuring balanced carrier injection, and minimizing losses at the interfaces. *η*_rad_ represents the fraction of radiative recombination for each electron‐hole pair and is thus directly related to the PLQY of emitters. *η*_out_ represents the escapability of the generated photon from the device, which is strongly affected by the device structure.

Careful selection and optimization of the various layers, based on their energy band alignment (see Figure 2c), are essential for achieving high efficiency, extended operational lifetime, and stable performance in PeLEDs. One of the main challenges in selecting appropriate HTLs and ETLs, particularly for blue LEDs, is finding compatible wide bandgap charge‐transport materials with the required energy levels and carrier mobilities to ensure low‐barrier and balanced charge transport.

## 3 Composition‐Controlled PeNCs for Blue Emission

**Figure 3:**
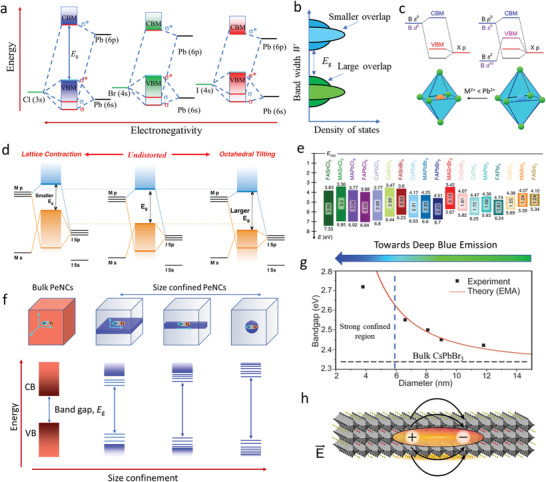
a) Schematic energy diagram of anion exchange CsPbX_3_ perovskites. b) The dispersion of band edges with overlapping. Reproduced with permission.^[^ ^98^ ^]^ Copyright 2016, Wiley‐VCH Verlag GmbH & Co. KGaA, Weinheim. c) Schematic energy diagrams of CsPbX_3_ perovskites with B‐site ns^0^ and ns^2^ np^0^ electronic configuration. Reproduced with permission.^[^ ^99^ ^]^ Copyright 2018, Wiley‐VCH Verlag GmbH & Co. KGaA, Weinheim. d) Band gap changes due to the distortion of the perovskite crystal structure. Reproduced with permission.^[^ ^84^ ^]^ Copyright 2017, American Chemical Society. e) Theoretically calculated band gap of the perovskite by changing the A, B, and X composition. Reproduced with permission.^[^ ^81^ ^]^ Copyright 2019, The Authors, published by Springer Nature. f) Illustration of dimension confinement, restricted degree of freedom of the exciton, and band gap opening in dimension‐confined PeNCs. g) Size‐dependent band gap energy of PeNCs and its comparison with the experimental value. Reproduced with permission.^[^ ^10^ ^]^ Copyright 2015, American Chemical Society. h) Dielectric confinement in PeNCs. Reproduced with permission.^[^ ^86^ ^]^ Copyright 2017, American Chemical Society.

Perovskite nanoparticles are known for their facile exchange of ions while preserving the crystal structures, which exert wide‐ranging effects on PeNC synthesis, processing, and surface functionalization. In the ABX_3_ perovskite structure, B‐site cation and X‐site anion significantly affect the band gap. In contrast, the A‐site cation indirectly affects the band edges by distorting the BX_6_^−^ inorganic networks. Therefore, the exchange of ions, specifically anions, in the BX_6_^−^ inorganic networks is the preferred method to synthesize blue PeNCs. Upon changing the X‐site anion from I^−^ to Br^−^ to Cl^−^, the band gap of the perovskite progressively increases, with a blue shift of the emission wavelength (**Figure** **3**a). This trend can be easily explained by the electronegativities of the elements, bond length, bond energy, orbital overlap, and bandwidth (*W*). X‐site anion is the major contributor to valence band maximum (VBM), and with an increase in the electronegativity of the X‐site anion, the p atomic orbital energy level of the X anion decreases, which downshifts VBM.^[^ ^81^, ^82^ ^]^

Furthermore, upon varying the X‐site anion from I^−^ to Br^−^ to Cl^−^, s and p atomic orbitals of the B‐site cation exhibit upward shifts. This shift is associated with reductions in the Pb‐X bond lengths, increasing electron confinement in the Pb atom. Consequently, this results in a higher energy level of the Pb atom, affecting the positions of VBM and conduction band minimum (CBM). Despite the elevations of the energy levels of the B‐site cation, the dominant downward shift of VBM induced by the X‐site anion widens the band gap, manifesting as a blue shift in the emission spectrum. Moreover, the bond strength and orbital overlap further elucidate the observed band gap widening and blue shift. With an increase in the electronegativity of the X‐site anion, the ionic character of the B‐X bond substantially increases. This increase in the ionic nature diminishes the overlap of atomic orbitals, thereby reducing *W* and resulting in a larger band gap (Figure 3b). This phenomenon underscores the critical interplay between anionic electronegativity, bond characteristics, and electronic band structure in determining the optical properties of colloidal PeNCs.

CBM of the ABX_3_ perovskite is predominantly derived from the s or d orbitals of the B‐site cation, specifically when this cation possesses an ns^0^ electronic configuration (e.g., Mn^2+^, Zn^2+^, and Cd^2+^). This configuration results in a substantial energy disparity between the occupied p orbitals of the X‐site anion and unoccupied s or d orbitals of the B‐site cation, leading to relatively large band gaps of perovskites. Additionally, the lattice experiences contraction due to the smaller size of the B‐site cation. Nevertheless, valence band dispersion, which is a critical factor affecting the electronic properties of perovskites, is significantly influenced by the electronic configuration of the B‐site cation. For instance, B‐site cations with ns^0^ electronic configurations exhibit low valence band dispersions as compared to those in the cases of cations with ns^2^ np° configurations, where significant orbital overlap occurs due to the high electron density (Figure 3c). Consequently, incorporating B‐site cations with ns^0^ electronic configurations into the perovskite crystal lattice results in a widened band gap and corresponding blue shift in the emission spectrum. This phenomenon is attributed to the low valence band dispersion and large band gap resulting from the less overlap between the electronic orbitals of the B‐site cation and X‐site anion. Furthermore, although the A‐site cation does not directly contribute to the formation of electronic band edges, the band gaps of ABX_3_ perovskites are indirectly influenced by the A‐site cation primarily due to the highly ionic nature of this cation. This indirect influence arises from the distortion of the ABX_3_ lattice caused by variations in the size of the A‐site cation. Lattice distortion includes lattice contraction and octahedral tilting of the BX_6_^−^ inorganic framework (Figure 3d), which decreases the connectivity and overlap of the atomic orbitals responsible for forming the band edges, leading to narrower *W* values and consequently a larger band gap. In contrast, lattice contraction tends to reduce the band gap by increasing the overlap of the orbitals. The Goldschmidt “tolerance factor” of the perovskite structure effectively describes this structural distortion.^[^ ^83^ ^]^ For instance, in ASnCl_3_ perovskites, the band gap increases with an increase in the size of the A‐site cation, whereas in ASnI_3_ perovskites, an opposite trend is observed (Figure 3e). These variations underscore the complex interplay between lattice distortions and electronic structure in determining the optical properties of perovskite materials.^[^ ^82^, ^84^ ^]^

## 4 Dimension‐Controlled PeNCs for Blue Emission

PeNCs demonstrate quantum and dielectric confinement effects when their sizes are decreased to the Bohr radius.^[^ ^10^, ^85^, ^86^ ^]^ Exciton binding energies of PeNCs with sizes below the Bohr radius are low, enlarging the band gap and causing a blue shift of the photoluminescence (PL) emission (Figure 3f).^[^ ^87^ ^]^ Confinement energy is calculated according to the following equation:^[^ ^10^ ^]^ Δ*E* = ℏ^2^π^2^/2m*r ^2^, where *r* is the particle radius and m* represents the reduced mass of the exciton. Theoretically calculated Bohr radii and exciton binding energies for CsPbCl_3_ (5 nm and 75 meV), CsPbBr_3_ (7 nm and 40 meV), and CsPbI_3_ (12 nm and 20 meV), respectively, are in excellent agreement with the corresponding experimental values.^[^ ^10^ ^]^

In mixed halide perovskites, ion segregation undermines device stability. However, in quantum‐confined PeNCs, blue emission can be achieved from a single perovskite component (CsPbBr_3_), resulting in high device stability without the problems of ion segregation.^[^ ^88^, ^89^, ^90^, ^91^ ^]^ Radiative recombination in PeNCs can also be increased by size confinement. In bulk PeNCs, radiative recombination involves bimolecular and monomolecular excitonic recombinations. In contrast, non‐radiative recombination is associated with Auger recombination and trap‐assisted monomolecular recombination. Due to the low exciton binding energies of bulk PeNCs, the generated excitons are prone to ionize into free charge carriers, facilitating Auger recombination. Moreover, trap‐assisted non‐radiative recombination predominates over bimolecular recombination at low charge carrier densities, resulting in low PLQY. Contrarily, in size‐ and dielectric‐confined PeNCs, the high exciton binding energies and restricted degrees of freedom prevent free migration of the generated excitons, leading to high PLQY (Figure 3g,h).

0D PeNCs with sizes 1–5 nm, known as QDs, exhibit size‐dependent optical properties, narrow emission lines, and near‐unity PLQYs. Quasi‐2D perovskite nanoplatelets (NPLs) with thicknesses ≈1.5–5.0 nm demonstrate tunable emissions ranging from green to blue, offering narrow emission peaks and high outcoupling efficiencies.^[^ ^92^, ^93^ ^]^ Perovskite nanowires (NWs)/nanorods (NRs) exhibit unique properties such as linearly polarized emission and anisotropic transition dipole moments (TDMs); however, due to their high ionic growth rates, controlling their sizes is challenging.^[^ ^94^, ^95^, ^96^, ^97^ ^]^ These diverse size‐confined perovskite structures hold promise for applications in optoelectronics, including LEDs, due to their tunable emissions, high PLQYs, and unique optical properties.

## 5 Colloidal Synthesis of PeNCs

PeNCs can be synthesized by many different methods^[^ ^100^, ^101^, ^102^, ^103^ ^]^ including colloidal synthesis,^[^ ^104^, ^105^ ^]^ ultrasonication,^[^ ^106^, ^107^ ^]^ mechanical mixing,^[^ ^108^, ^109^ ^]^ and high‐temperature solid‐state synthesis.^[^ ^110^, ^111^, ^112^ ^]^ Colloidal synthesis, the most developed method, allows precise control of the sizes,^[^ ^113^ ^]^ shapes,^[^ ^114^ ^]^ and compositions^[^ ^115^ ^]^ of PeNCs, leading to PeNCs with desired physical and chemical properties. It also allows mass production of nanoparticles with consistent sizes, shapes, and surface modifications.

**Figure 4:**
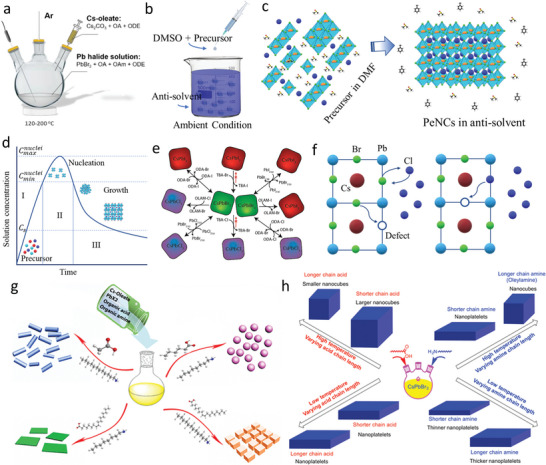
a) Schematic representation of the PeNCs synthesis by HI. Adapted with permission.^[^ ^66^ ^]^ b,c) Schematic illustration of the PeNCs synthesis by LARP. d) LaMer model of nucleation and crystal growth processes of PeNCs. Adapted with permission.^[^ ^145^ ^]^ e) Synthesis of blue PeNCs via the post‐treated anion exchange using different precursors. Reproduced with permission.^[^ ^146^ ^]^ Copyright 2015, American Chemical Society. f) Mechanism of defect induces anion exchange process. g,h) Synthesis of different shape control PeNCs by LARP and HI methods, respectively, controlling temperature and ligand size. Reproduced with permission.^[^ ^129^, ^147^ ^]^ Copyright 2016, American Chemical Society.

Colloidal synthesis of PeNCs is conducted using two main methods: hot injection (HI) and ligand‐assisted reprecipitation (LARP). In typical HI, PbX_2_ is dissolved in a nonpolar solvent such as octadecene (ODE) with oleic acid (OA) and oleylamine (OAm). Water is removed from the resulting solution under vacuum at 120 °C, followed by heating the reaction mixture to a desired temperature between 80 and 180 °C. PbX_2_ reacts with the solvent mixture, forming Pb‐oleate and oleylammonium halide (OAm‐X).^[^ ^116^ ^]^ Cs‐oleate precursor solution is prepared in a separate container by dissolving Cs_2_CO_3_ in ODE and OA at 100–120 °C under vacuum. Then, Cs‐oleate in ODE is hot‐injected into a mixture of Pb‐oleate and OAm‐X to trigger CsPbX_3_ formation (**Figure** **4**a).

LARP demonstrates advantages over HI in terms of simplicity and scalability. In LARP, the precursors of ABX_3_ are dissolved in a suitable solvent (N‐dimethylformamide, DMF) with long‐chain organic ligands, and then, the resulting solution is poured into an inferior solvent (such as toluene and hexane) followed by vigorous stirring.^[^ ^12^ ^]^ Under these conditions, the system undergoes spontaneous precipitation and crystallization until it reaches equilibrium. Mixing the precursor solution with the antisolvent immediately causes supersaturation, initiating the nucleation and development of PeNCs (Figure 4b,c). Despite their simplicities and scalabilities, PeNCs obtained by LARP are comparatively unstable due to the presence of numerous surface defect states, resulting in low PLQYs.^[^ ^117^ ^]^

Nucleation and crystal growth of PeNCs can also be described by the LaMer theory (Figure 4d).^[^ ^3^, ^118^ ^]^ According to this theory, in a solution mixture, monomer concentration increases over time until it reaches a supersaturation concentration (*C*_s_), denoted as stage I. Once the monomer concentration surpasses the minimum supersaturation concentration (*C*_min_), nucleation begins as the nucleation barrier is overcome, marking the onset of stage II. During this stage, crystals begin to grow, accompanied by further nucleation. When the monomer concentration decreases below the critical supersaturation value, nucleation ceases. However, during stage III, the preformed crystals continue to grow until the monomer concentration falls below the solubility limit. The nucleation rate is primarily determined during the nucleation stage, and nucleation controls the crystal size and distribution. Rapid burst nucleation is essential to achieve a homogeneous crystal size distribution. Due to the ionic natures of PeNCs, their nucleation barriers are low, facilitating rapid nucleation. Nucleation stage concludes with swift monomer depletion, allowing the nuclei to ideally grow without forming new nuclei. Consequently, HI can produce PeNCs with homogeneous size distributions. However, in HI, the Cs‐oleate precursor solution must be prepared in a separate flask and preheated (up to 120 °C) before injection. This requirement constrains the scalability of the production of PeNCs with uniform size distributions, narrow FWHM values, and high PLQYs.^[^ ^103^ ^]^

Blue‐emitting perovskites can also be synthesized through post‐synthetic anion exchange. Anion exchange involves supplying CsPbBr_3_ with an ample amount of Cl^−^ ion precursor in solution either at ambient or elevated temperatures. A large halide concentration gradient between the original perovskite surface and solution drives the diffusion of surface and grain boundary ions, creating defects. These defects eventually provide alternative low‐energy pathways for interior ion diffusion (Figure 4e,f).^[^ ^119^ ^]^

## 6 Dimensionality Control in Colloidal Synthesis

Sizes and shapes of PeNCs can be controlled by adjusting the reaction time and temperature, utilizing ligands and capping agents with varying chain lengths, modifying the precursors and their ratios, and selecting different solvents. In LARP, QDs are the major products when long‐chain ligands and capping agents are used. Zhang et al. synthesized MAPbX_3_ by LARP for the first time. Although micro‐sized non‐luminescent MAPbX_3_ particles were obtained without ligands, the employment of OAm and OA in the precursor solution led to QDs with high PLQYs of 70%.^[^ ^12^ ^]^ Long organic chains of the ligands attached to adjacent crystals repel each other, deterring crystal aggregation and enabling stable colloidal dispersion of nanocrystals in nonpolar organic solvents. Huang et al.^[^ ^120^ ^]^ observed that the particle size decreased with an increase in the amounts of OA and OAm. Cho et al. investigated the effects of the alkylamine chain length and relative amount of alkylamine on the sizes and shapes of PeNCs. They discovered that long‐chain acids favored the formation of small nanocubes. Long‐chain alkylamines at higher concentrations promoted NPL formation; in contrast, nanocubes were formed when short‐chain alkylamines were employed even at higher concentrations.^[^ ^121^ ^]^ Other studies also suggest that an increase in relative OAm/OA decreases the dimensionalities of PeNCs, and at higher OAm/OA, NPLs are formed.^[^ ^104^, ^122^, ^123^ ^]^ NPLs can be synthesized by LARP by adding a moderately polar solvent such as acetone, butanol, chlorobenzene, and ethyl acetate to the reaction mixtures.^[^ ^78^, ^124^ ^]^ Removal of the ligand from the edge, enabling 2D growth, is a possible reason for this. However, when temperature is increased from room temperature, the production of 3D nanostructures becomes more likely. Ahmed et al. observed a similar morphological development in the presence of pyridine. Pyridine can attach to the surface atoms, terminating the surface growth and driving the selective 2D growth of nanostructures at room temperature; in contrast, at elevated temperatures, nanocubes are formed.^[^ ^18^ ^]^ In LARP, the particle size increases when the temperature is slightly higher than room temperature.^[^ ^125^ ^]^ PLQY also increases at elevated temperatures due to the higher crystallinity of PeNCs. Ahmed et al. also observed that the sizes of the particles increased with an increase in the amount of precursor injected into the antisolvent, and at higher concentrations, nanocubes were formed.^[^ ^124^, ^126^ ^]^ Relative amount of precursor also affects the dimensions of PeNCs. Bohn et al. observed that with a decrease in Cs/Pb, 2D NPLs were formed.^[^ ^127^ ^]^ Almeida et al. observed similar results in the case of HI. They observed that at lower concentrations of the Cs‐oleate precursor, oleylammonium (RNH_3_^+^) competed with Cs^+^ and promoted the formation of low‐dimensional PeNCs through anisotropic growth.^[^ ^128^ ^]^ They also observed that both OA and OAm concentrations affected the formation of NPLs with a decrease in the concentration of the Cs‐oleate precursor solution. Figure 4g shows the schematic of the synthesis of PeNCs with different shapes by LARP via control of the chain lengths of the ligand and acid.

In HI, at low temperatures (80–120 °C), QDs are formed, whereas at high temperatures (140–200 °C), nanocubes are obtained. Sizes of QDs and NCs can be controlled by changing the chain length of the ligand. Whether nanocubes or NPLs will form also depends on the temperature and ligand chain length.^[^ ^129^ ^]^ Nanocubes are formed at high temperatures when long‐chain amines and acids (OA and OAm, respectively) are used. However, NPLs are formed when short‐chain amines are used. At low temperatures, NPLs are formed when short‐ and long‐chain acids and amines are employed.^[^ ^129^, ^130^ ^]^ Chain‐length dependency of the thicknesses of NPLs is also observed. The thinnest NPLs consisted of three perovskite unit cells and were prepared from shorter‐chain amines (Figure 4h). Shamsi et al. synthesized NPLs by injecting a Cs‐oleate precursor solution of only OA rather than a mixture of ODE and OA. They also synthesized NPLs using a mixture of short‐ and long‐chain acids and amines (octanoic acid (OctA), OA, octylamine (OctAm), and OAm) and observed that the thicknesses of NPLs decreased with an increase in the short‐chain acid and base ratios.^[^ ^131^ ^]^

Perovskite NRs/NWs can be synthesized by HI and LARP using a longer reaction time than that of nanocube formation.^[^ ^132^, ^133^, ^134^ ^]^ Temperature, precursor type, ratios, solvents, and ligands are important factors influencing the formation of NRs/NWs.^[^ ^124^, ^135^, ^136^, ^137^ ^]^ Dong et al. synthesized PeNCs with different morphologies at room temperature using acetone as an initiator solvent and CuBr_2_ and CoBr_2_ as precursors. NRs transformed into NPLs when excess CuBr_2_ was used, whereas they transformed into NWs when excess CoBr_2_ was used.^[^ ^136^ ^]^ Sun et al. observed a similar kind of polar solvent initiator effect using ethanol and NR formation through the self‐assembly of nanocubes.^[^ ^137^ ^]^ Teunis et al. synthesized NWs by heating a precursor solution in ODE, OAm, and OA in the presence of a small amount of DMF and injecting the resulting solution into acetone.^[^ ^138^ ^]^ Via HI, NRs/NWs can be obtained by adding excess short‐chain amines or acids. Excess short‐chain amines/acids block the seed surfaces, allowing growth to occur in only one dimension. Wang et al. synthesized NRs by conducting HI at 160 °C for 8 min using excess short‐chain amine (bis(2‐ethylhexyl)amine) with OAm. In contrast, when they used excess short‐chain acid, hexagonal NPLs were formed.^[^ ^139^ ^]^ However, Imran et al. synthesized NWs by conducting HI for 50 min at 120 °C using a short‐chain acid (hexanoic acid) and amine (OctAm) with OAm. They also discovered that the diameters of NWs decreased with an increase in the hexanoic acid content.^[^ ^140^ ^]^ Zhang et al. synthesized NWs by performing HI at 135 °C for 40–60 min using excess short‐chain amine (OctAm and OAm) without OA.^[^ ^141^ ^]^ NWs can be synthesized by conducting the reaction in the absence of ODE above 150 °C.^[^ ^142^ ^]^ Precursor‐dependent Pb‐free perovskite NRs have also been fabricated by the colloidal method.^[^ ^143^, ^144^ ^]^

## 7 Purification of PeNCs

Post‐synthesis work‐up is crucial to completely remove reactants, ligands, and solvents from the reaction mixture. Charge injection properties of the PeNC layer in the device can be improved by removing the nonconductive ligands through post‐synthesis purification; repetitive removal of the ligands increases film charge transport characteristics, thereby increasing the current densities of PeLEDs.^[^ ^148^, ^149^ ^]^ Furthermore, discarding the supernatants improves size distribution by removing small PeNCs, which removes the shoulder PL peak and decreases the FWHM of the PL peak. In HI, the perovskite solution is cooled down in an ice bath, which renders the solution mixture solid due to the low melting points of ODE, OA, and OAm, which are just below room temperature. Therefore, before centrifugation, the solution mixture should be fully liquefied using techniques including sonication and vortexing. Otherwise, the reactants and solvents will remain together with PeNCs. Ligands with long alkyl chains or aryl tails having hydrophilic amine groups enable appropriate dispersion of PeNCs in nonpolar solvents. Consequently, although nonpolar solvents act as antisolvents for perovskites, PeNCs remain completely dispersed in nonpolar solvents, such as hexane and toluene, due to the abovementioned surface ligands, rendering their separation from these solvents by centrifugation difficult.

Nevertheless, the ionic natures of PeNCs render them highly susceptible to polar solvents, which can lead to their decomposition and the creation of surface defects. Excessive removal of ligands by nonpolar solvents can increase non‐radiative recombination, thereby reducing PLQYs of PeNCs due to the formation of surface defects.^[^ ^19^, ^116^, ^149^ ^]^ Therefore, PeNCs should be washed with a mixture of nonpolar and moderately polar solvents. The ratio of solvent to antisolvent and centrifugation speed and time are crucial factors affecting the yields, purities, and PLQYs of PeNCs.

## 8 Blue‐Emitting Colloidal PeNCs and Their LEDs: The Current Status

### 8.1 Composition‐Controlled PeNCs

**Figure 5:**
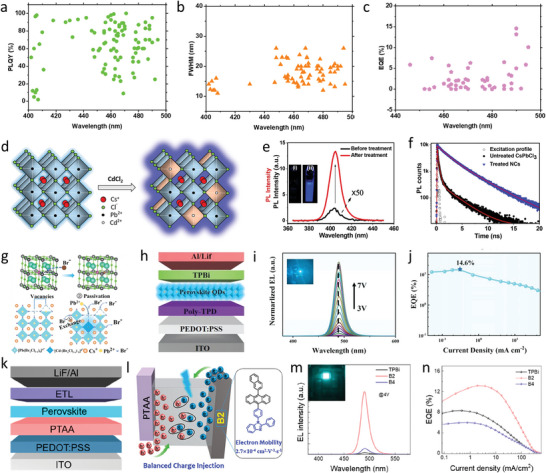
a) PLQY, b) FWHM, and c) EQE) of the composition control blue perovskite nanocubes. a–c) The data used for these figures are listed in Table 2 and Table S1 (Supporting Information). d) Representative Scheme for Cd^2+^‐doped CsPbCl_3_. e) PL intensity of treated and untreated CsPbCl_3_; The inset shows i) untreated CsPbCl_3_ and ii) treated CsPbCl_3_ under a UV lamp. f) PL decay dynamics (λ_ex_  = 375 nm, λ_PL_  = 406 nm) of CsPbCl_3_ NCs before and after the treatment with CdCl_2_. Reproduced with permission.^[^ ^153^ ^]^ Copyright 2018, American Chemical Society. g) Br^−^ enriched CsPb(Br*_x_*/Cl_1−_ *_x_*)_3_ NCs.  h) The device architecture. i) Electroluminescence spectra driven by voltages from 3 to 7 V; Inset image shows the luminescence of the device. j) EQE of the devices based on the exchange CsPb(Br*_x_*/Cl_1−_ *_x_*)_3_:Cd^2+^. Reproduced with permission.^[^ ^161^ ^]^ Copyright 2022, Wiley‐VCH GmbH. k,l) The device architecture and the structure B2 ETL, respectively. m) EL spectra of devices under 4 V. The inset is the photo of the perovskite QDs LED based on B2 lit up. n) EQE curves of PeLEDs on different ETLs. Reproduced with permission.^[^ ^28^ ^]^ Copyright 2023, American Chemical Society.

Perovskite nanocubes are the most examined PeNCs, whose emission wavelengths can be fine‐tuned by halide mixing. Br‐based perovskite nanocubes are extremely large to emit blue light, whereas Cl‐based perovskite nanocubes typically emit in the violet‐to‐deep‐blue range. Therefore, blue perovskite nanocubes are typically synthesized using mixed halides with different Cl^−^/Br^−^ values. PLQYs of pure Cl‐based perovskite nanocubes in the deep‐blue region are usually low. Low PLQYs at higher Cl^−^ ratios are due to the wide band gap and more trap‐assisted non‐radiative recombination. Lightweight and small size of Cl^−^ facilitate the formation of Cl^−^ vacancies in the perovskite structure, which trap charge carriers.^[^ ^150^, ^151^ ^]^ Various research groups have employed several passivation strategies to achieve near‐unity PLQYs from the deep‐blue to sky‐blue region (**Figure** **5**a). Furthermore, mixed halide perovskites with high color purities and narrow FWHM values can be achieved by fine‐tuning the ratio and types of halide ions in the perovskite composition. Additionally, FWHM values of mixed halide perovskites can be decreased by tuning the crystal structures via adjustment of the compositions of these perovskites (Figure 5b).

Passivating surface defects in perovskites with metal salts are widely used to enhance optoelectronic properties. Metal ions like Rb^+^, Ag^+^, Ca^2+^, Ni^2^⁺, Cu^2^⁺, Cd^2^⁺, and La^3+^ neutralize defects that trap charge carriers, reducing non‐radiative recombination and improving charge transport.^[^ ^152^, ^153^, ^154^, ^155^, ^156^, ^157^ ^]^ This surface passivation process boosts the PLQY, electroluminescence (EL) efficiency, and overall stability of perovskite devices. It also helps align energy levels, enabling better charge injection and recombination balance in PeLEDs, leading to higher efficiency, improved color purity, and increased device lifespan. Mondol et al. post‐treated CsPbCl_3_ nanocubes with Cd^2+^, achieving near‐unity PLQYs in a colloidal solution at an emission wavelength of 406 nm with high color purity (FWHM, 12 nm) (Figure 5d–f).^[^ ^153^ ^]^ Elemental compositions and structural information of the treated samples indicated that Cd^2+^ was easily doped into the crystal lattice without changing the sizes or shapes of NCs. These treated NCs exhibit exceptional PLQYs, high air stabilities, and photostabilities. FWHM values of mixed halide perovskites vary with the introduction of different halide ions, affecting the band gap energy and electronic structure.^[^ ^158^ ^]^ A‐site defects in perovskites can be effectively passivated using Rb⁺ ions due to their favorable chemical and structural properties. Rubidium belongs to the same alkali metal group as cesium and shares a similar ionic radius. This similarity allows Rb⁺ to integrate seamlessly into the perovskite crystal lattice without significantly disrupting the structure. Pan et al.^[^ ^159^ ^]^ employed a synergistic doping approach by introducing Rb^+^ and Ni^2+^ ions into the A and B sites of CsPb(Br_x_Cl_3‐x_) to create multiple‐cation blue PeNCs. This strategy resulted in a significant increase in PLQY to 86.7%. Rb^+^ ion doping allowed precise tuning of the emission wavelength while mitigating the negative effects of chlorine. Meanwhile, Ni^2+^ ion doping addressed halide vacancies and adjusted the energy band structure of the PeNCs, reducing the hole injection barrier and enhancing overall device performance.

Different organic ligands are also used to passivate the surface defects to enhance the PLQY of the PeNCs and EQE of the device. Zheng et al.^[^ ^160^ ^]^ achieved a high PLQY of 100% at an emission wavelength of 471 nm with high color purity (FWHM, 17 nm) by passivating CsPb(Br*_x_* Cl_1–_ *_x_*)_3_ perovskite nanocubes with *n*‐dodecylammonium thiocyanate (DAT). EQE of PeLED ITO/ poly(9,9‐dioctyl‐fluorene‐co‐n‐(4‐butylphenyl) diphenylamine (TFB)/ perfluorinated ionomer (PFI)/PeNC/3,3′,5,5′‐tetraphenylbenzidine (3TPYMB)/Liq/Al) fabricated using DAT‐doped CsPb(Br*_x_* Cl_1–_ *_x_*)_3_ was 6.3%, which is the highest EQE reported for mixed halide colloidal PeNCs in this region. The maximum luminance of the LED was 465 cd cm^−2^ with a half‐lifetime close to 99 s, considerably higher than those of pristine MHP QD LEDs (17 s), at a constant bias voltage of 4.5 V. High device stability is attributed to the low Cl vacancy density achieved using DAT, which diminishes ion migration by lowering the number of hopping sites. Lei et al.^[^ ^27^ ^]^ achieved an EQE of 3.2% at a constant current in the same emission region by passivating CsPb(Br_x_/Cl_1–x_)_3_ with didodecyldimethylammonium chloride (DDAC). They also achieved efficiencies of 4.0 and 9.0% for the sky‐blue emission peaks at 485 and 495 nm, respectively. The device comprised poly(triaryl)amine (PTAA) as the HTL and its overall configuration was ITO/poly(3,4‐ethylenedioxythiophene):polystyrenesulfonate PEDOT/ /poly(triarylamine) (PTAA) /PeNC/1,3,5‐Tris (1‐phenyl‐1H‐benzimidazol‐2‐yl)benzene (TPBi)/LiF/Al. PLQYs of DDAC‐treated CsPb(Br_x_/Cl_1–x_)_3_ were measured to be 75, 83, and 41% with FWHM of 14 nm at the emission wavelengths of 495, 485, and 470 nm, respectively. Low PLQY in the blue region might be due to the removal of surface ligands by excessive doping of CsPb(Br_x_/Cl_1–x_)_3_ with DDAC and the introduction of deep‐level defects by Cl ions. Maximum luminances of 780 and 662 cd m^−2^ were obtained at the emission wavelengths of 495 and 485 nm, respectively. Zhu et al.^[^ ^161^ ^]^ achieved a breakthrough efficiency using Cd^2+^‐doped perovskite nanocubes subjected to surface passivation with PbBr_2_ and di‐n‐decyl dimethylammonium bromide (DDAB). Br‐enriched surface effectively passivated vacancy defects, improving PLQY to 95% with FWHM of 20 nm, at an emission wavelength of 490 nm. Film processed with controlled PeNCs exhibited a crack‐free morphology with fewer defects and high carrier transport, resulting in high EQEs of 14.6% in LEDs with a device structure of ITO/PEDOT/Poly‐TPD/PeNCs/TPBi/LiF/Al (Figure 5g–j).

Although PLQYs of PeNCs can approach unity, EQEs of LEDs do not correspondingly increase due to various factors influencing device performance. Figure 5c shows that EQE increases with an increase in the emission wavelength, indicating that the charge injection in the device is limited due to the energy level mismatch between the perovskite layer and the charge transport layers in the blue PeLEDs. To eliminate this limitation, Yuan et al.^[^ ^28^ ^]^ fabricated an LED with a similar device structure except for the use of a tailor‐made small organic molecule as electron‐transporting material (ETM) named B2 using organic‐inorganic mixed halide perovskites (FA_1−x_Cs_x_PbBr_1−x_Cl_x_) and achieved a high efficiency. Because of the high electron mobility of B2, charge injection in the perovskite QD LEDs was well‐balanced, and non‐radiative recombination at the interface was significantly suppressed. Consequently, blue LEDs with perovskite QDs and B2 as ETM exhibited promising EQEs of 13.17% with turn‐on voltages of 2.2 V and maximum luminances of 8656.67 cd·m^−2^ (Figure 5k–n).

Although organic ligands are often used to passivate the surface defects of the PeNCs and enhance the PLQY, their insulating nature limits the EQE of PeLEDs by hindering charge injection between the perovskite layer and the charge transport layers. To overcome this, long‐chain ligands are replaced with short‐chain ones to improve charge injection and EQE. Qin et al.^[^ ^162^ ^]^ proposed a co‐regulation strategy using double short‐chain molecules of tetraoctylammonium fluoride (TOAF) and tetraethylammonium perfluorooctanesulfonate (TEA‐PFOS) to enhance the regrowth of blue Cs_x_Rb_1−x_Pb(Cl_y_Br_1−y_)_3_ PeNCs. These short‐chain ligands, with higher adsorption energy, replaced long‐chain insulating OAm and OA, allowing PeNCs to grow from ≈8 to ≈18 nm and significantly reducing defect‐state density by 3–5 times. This resulted in highly efficient blue PeNCs with a PLQY of nearly 100%. Additionally, the PeNCs showed lower insulating ligand density and enhanced charge injection properties. Consequently, the deep‐blue PeLEDs achieved a maximum external quantum efficiency (EQE) of 5.7% at the CIE coordinates of (0.148, 0.032). While ligand exchange is a promising technique for enhancing stability, few studies have compared the effectiveness of different ligand ion pair combinations. Li et al.^[^ ^163^ ^]^ investigated the effectiveness of various ion pairs on enhancing device performance and stability and discovered that cetrimonium tosylate (CTTS) effectively reduces surface defects in PeNCs while increasing exciton binding energy. This led to a significant improvement in PLQY, from 31% to 65.7%, without any spectral shift. By optimizing ligand‐exchanged PeNCs, they achieved a highly efficient and stable pure blue LED with a peak EQE of 7.45% and a T_50_ lifetime of 61 minutes. **Table** **2** and Table S1 (Supporting Information) summarize the optoelectronic properties and LED performances of composition‐controlled perovskite nanocubes.

**Table 2:** Summary of the optoelectronic properties of perovskite nanocubes and performances of perovskite nanocube‐based LEDs.

Composition of Nanocubes	Synthesis Method	Defect treatment	Device Structure	PL Wavelength [nm]	PLQY [%]	FWHM [nm]	EQE [%]	PL Lifetime [ns]/^*^EL Lifetime [T_50_] [s]	Year	Ref
CsPbCl_3_	HI	Water & TMOS	‐	410	78.5	‐	‐	‐	2018	[164]
CsPbCl_2_Br	HI	NaBF_4_ or NH_4_BF_4_	‐	427	90	‐	‐	‐	2018	[165]
CsPbCl_1.5_Br_1.5_	HI	NaBF_4_ or NH_4_BF_4_	‐	442	95	‐	‐	‐	2018	[165]
CsPbBr_2_Cl	HI	NaBF_4_ or NH_4_BF_4_	‐	458	96	‐	‐	‐	2018	[165]
CsPbCl_3_:Ni^2+^	HI	Ni^2+^‐doped	‐	405	96.5	‐	‐	18.39	2018	[152]
CsPbCl_2.4_Br_0.6_:Ni^2+^	HI	Ni^2+^‐doped	‐	418	92.0	‐	‐	15.56	2018	[152]
CsPbCl_2_Br:Ni^2+^	HI	Ni^2+^‐doped	‐	436	93.1	‐	‐	11.31	2018	[152]
Cs(Pb/Cd)Cl_3_	HI	Cd^2+^‐doped	‐	406	98	12	‐	4.20	2019	[153]
CsPbBr*_x_* Cl_3−_ *_x_*:Y^2+^	HI	Y^2+^‐doped	‐	464	75	‐	‐	3.7	2018	[166]
CsPbBr*_x_* Cl_3−_ *_x_*:Ni^2+^	HI	Ni^2+^‐doped	‐	466	85	‐	‐	4.2	2018	[166]
CsPbBr*_x_* Cl_3−_ *_x_*:Cu^2+^	HI	Cu^2+^‐doped	‐	430	92	14	‐	4.3	2019	[154]
CsPbBr*_x_* Cl_3−_ *_x_*:Cu^2+^	HI	Cu^2+^‐doped	‐	460	98	‐	‐	3.6	2019	[154]
CsPbBr*_x_* Cl_3−_ *_x_*:Cu^2+^	HI	Cu^2+^‐doped	‐	460	92.6	‐	‐	‐	2019	[167]
CsPb_1–_ *_x_* Cu*_x_* (Br/Cl)_3_	HI	Cu^2+^‐doped	‐	448	78	26	‐	4.6	2019	[168]
CsPb_1–_ *_x_* Cu*_x_* (Br/Cl)_3_	HI	Cu^2+^‐doped	‐	455	80	23	‐	5.0	2019	[168]
(Rb/Cs)PbBr*_x_* Cl_3−_ *_x_*	LARP	Rb^+^‐doped	‐	468	89	26	‐	2.3	2019	[155]
CsPbBr*_x_* Cl_3−_ *_x_*:Ni^2+^	LARP	Ni^2+^‐doped	‐	470	89	‐	‐	6.2	2020	[169]
CsPbBr*_x_* Cl_3−_ *_x_*:Ni^2+^	LARP	Ni^2+^‐doped	‐	480	87	‐	‐	‐	2020	[169]
CsPbBr*_x_* Cl_3−_ *_x_*:La^3+^	HI	La^3+^‐doped	‐	464	68.3	23.8	‐	7.83	2020	[156]
CsPbBr*_x_* Cl_3−_ *_x_*:La^3+^	HI	La^3+^‐doped	‐	469	75.3	22.9	‐	8.6	2020	[156]
CsPbBr*_x_* Cl_3−_ *_x_*:La^3+^	HI	La^3+^‐doped	‐	448	50.36	22.8	‐	7.08	2020	[156]
CsPbBr*_x_* Cl_3−_ *_x_*:Ag^+^	HI	Ag NPs‐doped	‐	465	77	‐	‐	13.02	2021	[170]
CsPbCl_3−x_Br_x_	LARP	No	‐	438	69.2	15	‐	1.95	2021	[171]
CsPbCl_3−x_Br_x_	LARP	No	‐	456	89.8	17	‐	2.23	2021	[171]
CsPbCl_3−x_Br_x_	LARP	No	‐	470	85.4	16	‐	5.18	2021	[171]
CsPbCl_3−x_Br_x_	LARP	No	‐	488	86.1	18	‐	10.31	2021	[171]
Cs_x_Rb_1‐x_Pb(Cl_y_Br_1‐y_)_3_	HI	No	‐	447	58.67	14.6	‐	6.58	2024	[162]
Cs_x_Rb_1‐x_Pb(Cl_y_Br_1‐y_)	HI	TOAF	‐	459	81.81	15.8	‐	10.42	2024	[162]
CsPbBr_x_Cl_3‐x_	HI	No	ITO/PEDOT/TFB/PFI/PeNCs/TPBi/LiF	488	‐	‐	1.41	‐	2018	[76]
(Rb/Cs)PbBr_x_Cl_3−x_:Ni^2+^	HI	Rb^+^, Ni^2+^‐doped	ITO/PEDOT:PSS/ poly‐TPD/PVK/PeNCs/TPBi/LiF/Al	466	86.7	15	2.14	5.72	2020	[159]
CsPbBr*_x_* Cl_3−_ *_x_*:Ni^2+^	LARP	Ni^2+^‐doped	ITO/PEDOT:PSS/ TFB/PFI/PeNCs/TPBi/LiF/Al	469	89		2.4	11.9	2020	[169]
CsPbBr*_x_* Cl_3−_ *_x_*:La^3+^	HI	La^3+^‐doped	ITO/PEDOT:PSS/PVK/PeNCs/TPBi/LiF/Al	478	78.7	22.4	2.17	‐	2020	[156]
CsPbBr*_x_* Cl_3−_ *_x_*:La^3+^	HI	La^3+^‐doped	ITO/PEDOT:PSS/PVK/PeNCs/TPBi/LiF/Al	488	82.2	20.8	3.25	‐	2020	[156]
PEA‐CsPb(Cl_x_/Br_1‐x_)_3_	HI	PEACl	ITO/PEDOT/PVK/PeNCs/TmPyPB/LiF/Al	465	74.5	19	0.92	‐	2020	[172]
PEA‐CsPb(Cl_x_/Br_1‐x_)_3_	HI	PEACl	ITO/PEDOT/PVK/PeNCs/TmPyPB/LiF/Al	468	80.3	20	1.53	‐	2020	[172]
PEA‐CsPb(Cl_x_/Br_1‐x_)_3_	HI	PEACl	ITO/PEDOT/PVK/PeNCs/TmPyPB/LiF/Al	470	82.4	21	2.15	^*^23.6	2020	[172]
CsPb(BrCl_1‐x_)_3_	HI	DAT	ITO/TFB/PFI/PeNCs/3TPYMB/Liq/Al	471	100	17	6.3	^*^99	2020	[160]
CsPb(Br_x_Cl_1−x_)_3_:Cd^2+^	HI	Cd^3+^‐doped, DDAB	ITO/PEDOT/Poly‐TPD/PeNCs/TPBi/LiF/ Al	490	95	20	14.6	21.08/^*^1020	2022	[161]
FA_1–_ *_x_* Cs*_x_* PbBr_1–_ *_x_* Cl*_x_*	LARP	DDAC	ITO/PEDOT:PSS/PTAA/PeNCs/B2/LiF/Al	465	‐	20	2.01		2023	[28]
FA_1–_ *_x_* Cs*_x_* PbBr_1–_ *_x_* Cl*_x_*	LARP	DDAC	ITO/PEDOT:PSS/PTAA/PeNCs/B2/LiF/Al	490	‐	20	13.17	^*^1200	2023	[28]
CsPb(Br*_x_* Cl_1−_ *_x_*)_3_	HI	DDAC	ITO/PEDOT/PTAA/PeNCs/TPBi/LiF/Al	470	41	14	5.1	51.75	2023	[27]
CsPb(Br*_x_* Cl_1−_ *_x_*)_3_	HI	DDAC	ITO/PEDOT/PTAA/PeNCs/TPBi/LiF/Al	485	83	14	6.5	‐	2023	[27]
CsPb(Br*_x_* Cl_1−_ *_x_*)_3_	HI	DDAC	ITO/PEDOT/PTAA/PeNCs/TPBi/LiF/Al	495	75	14	10.1	42/^*^43200	2023	[27]
CsPb(Br_1‐x_C_x_)_3_/ZnS	HI	ZnS shell	ITO/PEDOT/TFB/PeNCs/TPBi/ LiF/Al	451	41.2	17.8	1.32	^*^1192	2023	[21]
Cs(Pb,Cd)Br_3_	HI	Cd‐doped	ITO/PEDOT/polyTPD/PVK/PeNCs/ TPBi/LiF/Al	485	89.9	19.5	0.04		2024	[173]
CsPbBr_1.8_Cl_1.2_	HI	CTTS	ITO/PEDOT:PSS/Poly‐TPD/PVK/PeNCs/TPBi/LiF/Al	455	65.7	‐	7.45	^*^3660	2024	[163]
Cs_0.5_Rb_0.5_PbBr_3_	LARP	Rb‐doped	ITO/PEDOT/Poly‐TPD/ PeNCs/TPBi/LiF/Al	494	70	26	5.9	^*^882	2024	[174]
Cs_x_Rb_1‐x_Pb(Cl_y_Br_1‐y_)_3_	HI	TOAF, TEA‐PFOS	ITO/PEDOT /Poly‐TPD/PeQDs/TPBi/LiF/Al	462	99.26	14.4	5.7	19.13	2024	[162]
CsPb(Br*_x_* Cl_1−_ *_x_*)_3_	HI	ETFA	ITO/PEDOT /TFB/perovskite NCs/TPBi/LiF/Al	463	80	20	4.14	39.43/^*^316	2024	[175]
CsPb(Br*_x_* Cl_1−_ *_x_*)_3_	HI & Ion exchange	Ca^2+^ ‐doped	ITO/PEDOT /PVK/PQDs/TPBi/LiF/Al	446	80.3	‐	5.88	‐	2024	[157]

TMOS = tetramethyl orthosilicate, PEACl = Phenethylammonium chloride, DAT = n‐dodecyl ammonium thiocyanate, DDAC = di‐n‐decyl dimethylammonium chloride, DDAB = di‐n‐decyl dimethylammonium bromide.

### 8.2 Dimension‐Controlled PeNCs

**Figure 6:**
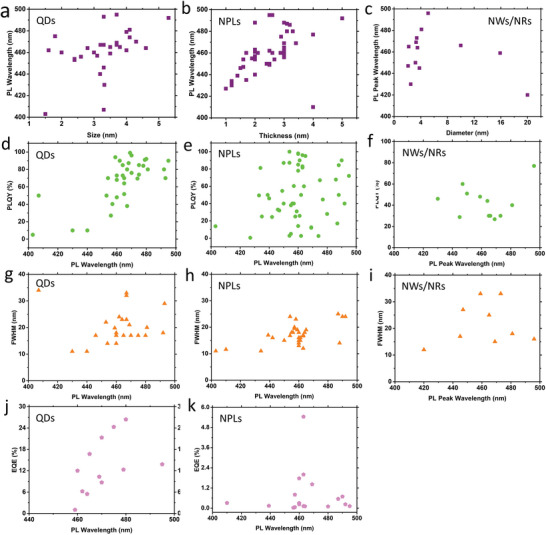
Optoelectronic properties of dimension‐controlled PeNCs. Size‐dependent PL emission wavelength of a) QDs, b) NPLs, and c) NWs/NRs, respectively. Size‐dependent PLQY of d) QDs, e) NPLs, and f) NWs/NRs, respectively. Size‐dependent FWHM of g) QDs, h) NPLs, and i) NWs/NRs, respectively. Size‐dependent EQE of j) QDs and k) NPLs, respectively. (a, d, g, j): The data used for these figures are listed in Table 3 and Table S2 (Supporting Information). (b, e, h, k): The data used for these figures are listed in Table 4 and Table S3 (Supporting Information). (c, f, i): The data used for these figures are listed in Table 5.

**Figure** **6** shows the optoelectronic properties of size‐controlled PeNCs. Pure CsPbBr_3_ perovskite QDs with sizes below 3.5 nm emit in the deep‐blue region, whereas those with sizes above 3.5 nm emit in the blue‐to‐sky‐blue region. However, chloride‐based pure and mixed halide perovskite QDs with a size above 3.5 nm emit in the violet region. Perovskite NPLs are other size‐confined nanostructures whose dimensionalities are controlled in one direction and are widely used to investigate blue LEDs. Thicknesses of NPLs can be controlled from a single layer to multiple layers. With an increase in the NPL thickness, the emission wavelength increases. Perovskite NPLs with thicknesses of 3.0 nm emit in the deep‐blue‐to‐blue region, whereas those with thicknesses ranging from 3.0 to 5.0 nm emit in the sky‐blue region. For perovskite NWs/NRs, blue emission occurs when the diameters of these NWs/NRs are controlled in the range of 2.0–4.0 nm. However, NWs/NRs with chloride compositions and broader diameters can emit in the deep‐blue‐to‐violet region. Figure 6a–c show the size‐dependent PL emission wavelength of QDs, NPLs, and NWs/NRs, respectively.

**Figure 7:**
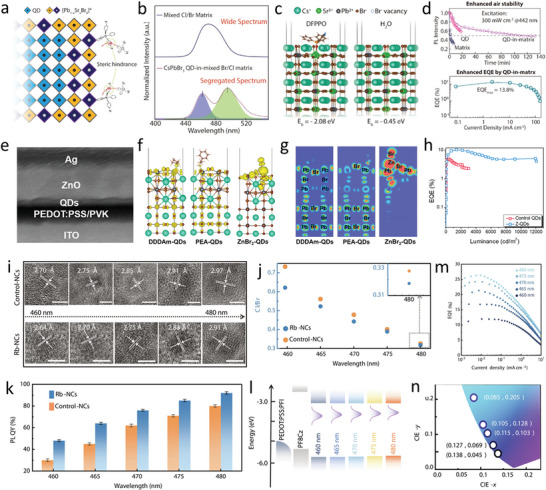
a) Passivated QDs‐in‐matrix structure. b) PL spectra of the mixed Br/Cl perovskite matrix and CsPbBr_3_ QD‐in‐mixed Br/Cl perovskite matrix solids. c) DFT calculated the binding energy of DFPPO and H_2_O with the Sr^2+^–perovskite surface. d) Stability and EQE of the device. Reproduced with permission.^[^ ^177^ ^]^ Copyright 2022, American Chemical Society. e) STEM cross‐section view image of a device with Z‐QDs. f,g) DFT calculation of electronic charge density with two additional electrons under an external electronic field near the valence band (yellow) for DDDA‐QDs, PEA‐QDs, and ZnBr_2_‐QDs. h) EQE of the PeLEDs versus luminance. Reproduced with permission.^[^ ^90^ ^]^ Copyright 2022, American Chemical Society. i) Crystal lattice fringes of control QDs (top row) and Rb‐doped QDs (bottom row) are shown. Scale bar: 5 nm. j) Molar ratios of Cl to Br in both control QDs and Rb‐doped QDs are presented. k) PLQYs of control QDs and Rb‐doped QDs, exhibiting minor differences in Cl content, are compared. l) A schematic energy level diagram illustrates the layers of PEDOT:PSS, PF8Cz, and the nanocrystals. m) EQE–current density (*J*) curves of PeLEDs utilizing Rb‐doped QDs are displayed. n) Chromaticity coordinates of PeLEDs are shown for the 460–480 nm wavelength range. Reproduced with permission.^[^ ^25^ ^]^ Copyright 2024, AAAS.

PLQYs of dimension‐controlled PeNCs with passivated surface defects are likely to approach unity (Figure 6d–f). FWHM values of size‐controlled PeNCs depend on the size distributions of PeNCs. Variations in NC size can lead to spectral broadening and color impurities due to differences in band gap energies of the NC. High‐color‐purity blue emission can be achieved from size‐controlled PeNCs, and most of these PeNCs exhibit FWHM values within 12–25 nm (Figure 6g–i). Yitong et al.^[^ ^176^ ^]^ achieved precise size control of CsPbBr_3_ QDs with exceptional ensemble uniformity by utilizing thermodynamic equilibrium. The QDs, with a uniform size of 3.7 nm, exhibited a high PLQY of 80–90% and a FWHM of 23 nm. As the QD size increased to 5.3 nm, the FWHM became narrower, due to the more precise size control of the QDs and thus reduced size distribution. QDs also demonstrate outstanding EQEs in the blue‐to‐sky‐blue region (Figure 6j). As noted earlier, long‐chain insulating ligands limit PeLED efficiency. To address this, Dong et al.^[^ ^89^ ^]^ removed the long chain OAm and coated the QDs with a Br‐rich outer shell, which stabilized the QDs and resulted in high PLQYs. After removing the long‐chain ligand, isopropylammonium bromide was introduced into QDs to form a Br‐rich outer shell. Subsequently, NaBr was used to eliminate the remaining organic ligands and passivate the QD surfaces. Bipolar‐shell‐protected QDs effectively suppressed anion exchange and electron quenching, achieving PLQYs of 91% at an emission wavelength of 479 nm with FWHM of 20 nm. LEDs incorporating QDs exhibited low turn‐on voltages of 2.8 V and high EQEs of 12.3%, and the device configuration was ITO/PEDOT:PSS/PTAA/QDs/TPBi/LiF/Al. In their following study, they passivated bipolar‐shell‐protected QDs with bis(4‐fluorophenyl)phenylphosphine oxide (DFPPO) and incorporated Sr^2+^ dopants into the perovskite matrix. PLQY of the QD‐in‐mixed Sr/Pb perovskite matrix was similar to those of bipolar‐shell‐protected QDs at an emission wavelength of 495 nm. However, the DFPPO‐passivated and Sr^2+^‐doped perovskite matrix enhanced the stabilities and performances of LEDs, reaching EQEs and luminances of up to 13.8% and 6113 cd m^−2^, respectively. The device structure was the same as that in their previous study except for the use of perfluorinated resin solution as HTL with PEDOT:PSS. Perovskite QD‐in‐matrix solids demonstrate type‐I band alignments and lattice matching between QDs and matrix. The high stability of the device was attributed to the DFPPO molecules, which exhibited strong binding with Sr^2+^ and provided sufficient steric hindrance to block H_2_O (**Figure** **7**a–d).

Liu et al. synthesized high‐PLQY (96%) perovskite QDs, and the corresponding device with the structure ITO/PEDOT/PVK/PeNC‐ZnO/Ag achieved 8.7% EQE at 470 nm. Turn‐on voltage was 3.0 V with a maximum luminance of 11 134 cd m^−2^. Surfaces of perovskite QDs were passivated with didodecylamine (DDDA). After passivation, difunctional ZnO (D‐ZnO) NCs were mixed with perovskite QDs for film formation.^[^ ^70^ ^]^ D‐ZnO ligand not only filled the O vacancy (O_V_) defects in ZnO and repaired the perovskite emission layers damaged by the D‐ZnO solution solvent, but also reduced the conductivity of the ETL and increased the energy barrier for electron injection. This led to a balanced charge distribution in the EMLs of PeLEDs. In their following study, perovskite QD surfaces were passivated with HBr, DDDA, and phenylethylamine, and then, the long‐chain ligand was removed by ZnBr_2_. PLQYs of ZnBr_2_‐treated QDs (Z‐QDs) were 99% with FWHM of 21 nm at an emission wavelength of 469 nm. Surface ligand modification of QDs with ZnBr_2_, which acts as a capacitor, alleviates charge accumulation and reduces the exciton binding energies of QDs, suppressing the Auger recombination, resulting in substantially lower efficiency roll‐offs of PeLEDs, and the device with a configuration of ITO/PEDOT/poly(N‐vinylcarbazole) (PVK)/PeNC/ZnO QDs/Ag demonstrates an outstanding EQE of 10.3% (Figure 7e–h).^[^ ^90^ ^]^ **Table** **3** and Table S2 (Supporting Information) summarize the optoelectronic properties of perovskite QDs and the performances of perovskite QD‐based LEDs, respectively.

**Table 3:** Summary of the optoelectronic properties of perovskite QDs and performances of perovskite QDs‐based LEDs.

Composition of PeNCs	Size [nm]	Synthesis Method	Defect treatment	Device Structure	PL Peak Wavelength [nm]	PLQY [%]	FWHM [nm]	EQE [%]	PL Lifetime [ns]/^*^EL Lifetime [T_50_] [s]	Year	Reference
MAPbCl_0.6_Br_2.4_	3.3	LARP	No	‐	493	70	29	‐	12	2015	[12]
MAPbBr_3_	1.8	LARP	No	‐	475	74	‐	‐	17	2015	[125]
CsPbBr_3_	3.0	One pot	No	‐	460	68	18	‐	‐	2017	[184]
CsPbBr_3_	3.8	HI	OAmBr	‐	465	70	‐	‐	5.24	2018	[113]
CsPbBr_3_	3.7	HI	Zn^2+^‐doped	‐	467	80‐90	23	‐	‐	2018	[176]
CsPbBr_3_	4.1	HI	Zn^2+^‐doped	‐	481	80‐90	20	‐	‐	2018	[176]
CsPbBr_3_	5.3	HI	Zn^2+^‐doped	‐	492	80‐90	18	‐	‐	2018	[176]
CsPbBr_3_:Sb^3+^	2.0	LARP	Sb^3+^‐doped	‐	460	73	14	‐	4.5	2019	[185]
CsPbBr_3_	2.8	LARP	No	‐	468	87.20	‐	‐	12.24	2021	[186]
CsPbBr_3_	1.6	LARP	No	‐	462	‐	‐	‐	‐	2021	[187]
FAPbBr_3_	3.19	LARP	No	‐	440	10	11	‐	‐	2024	[188]
MAPbBr_3_	3.32	LARP	No	‐	430	10	11	‐	‐	2024	[188]
MAPbBr_3_	2.4	HI	No	ITO/PEDOT/PVK/QDs/TPBi/CsF/Al	454	70	14	‐	31.4	2018	[189]
CsPbBr3	4.0	HI	IPABr/NaBr	ITO/PEDOT:PSS/PTAA/QDs/TPBi/LiF/Al	479	91	‐	12.3	^*^1200	2020	[89]
CsPb_1–_ *_x_* Sr*_x_* Br_3_	3.7	HI	Sr^2+^‐doped	ITO/PEDOT:PSS;PFI/QDs mixed matrix/TPBi/LiF/Al	495	90	‐	13.8	‐	2022	[177]
CsPbBr_3_/ZnO	4.3	HI	HBr & DDDA	ITO/PEDOT/PVK/QDs‐ZnO/Ag	470	96		8.7	^*^75600	2022	[70]
CsPbBr_3_	3.7	HI	HBr & DDDA	ITO/PEDOT/PVK/QDs/ZnO QDs/Ag	469	99	21	10.3	^*^90 000	2023	[90]
CsPbBr_3_	4.6	LARP	No	ITO/PEDOT:PSS/PVK:TFB/QDs/TPBi/LiF/Al	464	73	23	5.46	^*^840	2024	[190]
CsPbBr_3_	4	HI	BnBr & PEA	ITO/PEDOT:PSS/Oxe‐DCDPA/QDs/CN‐T2T/LiF/Al	462	90	24	6.2	7.34/^*^201	2024	[79]
CsPbBr_3_	3.5	LARP	No	ITO/PEDOT/TFB/PEABr/QDs/PO‐T2T/LiF/Al	459	94	19.8	1.0	‐	2024	[191]
(Rb/Cs)Pb(Br*_x_*/Cl_1−_ *_x_*)_3_	7.8	LARP	DBSA	ITO/PEDOT:PSS/PFI/PF8Cz/QDs/TPBi/LiF/Al	460	48	17	12.0	48.4	2024	[25]
(Rb/Cs)Pb(Br*_x_*/Cl_1−_ *_x_*)_3_	8.0	LARP	DBSA	ITO/PEDOT:PSS/PFI/PF8Cz/QDs/TPBi/LiF/Al	465	64	17	16.7	45.1	2024	[25]
(Rb/Cs)Pb(Br*_x_*/Cl_1−_ *_x_*)_3_	8.5	LARP	DBSA	ITO/PEDOT:PSS/PFI/PF8Cz/QDs/TPBi/LiF/Al	470	76	17	21.3	42.7	2024	[25]
(Rb/Cs)Pb(Br*_x_*/Cl_1−_ *_x_*)_3_	8.2	LARP	DBSA	ITO/PEDOT:PSS/PFI/PF8Cz/QDs/TPBi/LiF/Al	475	85	17	24.3	38.7	2024	[25]
(Rb/Cs)Pb(Br*_x_*/Cl_1−_ *_x_*)_3_	8.2	LARP	DBSA	ITO/PEDOT:PSS/PFI/PF8Cz/QDs/TPBi/LiF/Al	480	92	17	26.4	35.1	2024	[25]

BnBr = benzoyl bromide, PEA = phenethylamine, PO‐T2T = 2,4,6‐Tris[3‐(diphenylphosphinyl)phenyl]‐1,3,5‐triazine.

Jang et al.^[^ ^79^ ^]^ introduced a “QD‐in‐solid solution” approach to achieve deep‐blue emission with high efficiency. In this approach, QDs are dispersed within a hole‐transporting solid matrix, *N,N*′‐dicarbazolyl‐3,5‐benzene (mCP), forming a “solid solution” that prevents the spectral redshift commonly observed in film states while maintaining the intrinsic deep‐blue emission of 4 nm perovskite QDs. The redshift in the EL spectrum typically results from electronic coupling and energy transfer between closely spaced QDs. By increasing the spatial separation between the QDs in the solid solution, these interactions are minimized, effectively preserving the deep‐blue emission without the undesirable redshift. The material design also avoids low‐energy peaks caused by exciplex formation, often seen when QDs are dispersed in organic semiconductors. The deep HOMO level (6.0 eV) of the mCP helps to create a greater energy barrier between the QDs and the mCP. As a result, the interaction between the QDs and the mCP is minimized, which reduces the likelihood of forming exciplexes. The PeLEDs with device structure ITO/PEDOT:PSS/3,5‐di‐9H‐carbazol‐9‐yl‐*N,N*‐bis[4‐[[6‐[(3‐ethyl‐3‐oxetanyl)methoxy]hexyl]oxy]phenyl]benzenamine (Oxe‐DCDPA)/QDs‐mCP/1,3,5‐triazine‐2,4,6‐triyl) tris(([1,1′‐biphenyl]‐3‐carbonitrile)) (CN‐T2T)/LiF/Al achieved deep‐blue EL emission with CIE coordinates of (0.138, 0.058) and a peak EQE of 6.2%.

Gao et al.^[^ ^25^ ^]^ conducted an in‐depth study on the impact of Cl content on the performance of blue PeLEDs by developing a series of CsPb(Br_x_/Cl_1‐x_)_3_ QDs. They found that the EQE of blue PeLEDs, with emission peaks at 460, 465, 470, 475, and 480 nm, scales linearly with the PLQY. The PLQY of these nanocrystals is highly sensitive to Cl content, due to the formation of deep‐level trap states. To address this, they reduced the Cl/Br ratio and introduced Rb at the A‐site of the perovskite. The introduction of the Rb passivated the defect sites and enhanced the PLQY without altering emission wavelengths. This approach led to blue PeLEDs with the device structure ITO/PEDOT:PSS/PFI/poly((9,9‐dioctylfluorenyl‐2,7‐diyl)‐alt‐(9‐(2‐ethylhexyl)‐carbazole‐3,6‐diyl) (PF8Cz)/QDs/TPBi/LiF/Al, showing high efficiencies with peak EQEs of 12.0% at 460 nm, 16.7% at 465 nm, 21.3% at 470 nm, 24.3% at 475 nm, and 26.4% at 480 nm. The maximum luminance reaches 1601, 1175, 635, 430, and 255 cd m^−2^ for PeLEDs at the corresponding EL peaks. This study highlights Cl‐defect control as a crucial strategy for high‐efficiency blue PeLEDs (Figure 7i–n).

**Figure 8:**
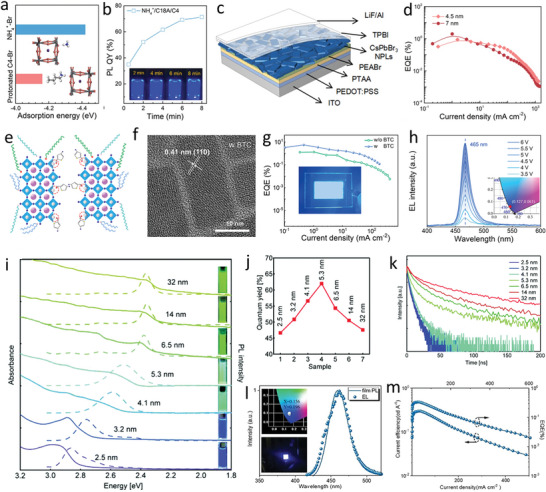
a) Adsorption energy of protonated C4–Br and NH_4_^+^–Br. The inset shows a protonated C4‐ and NH_4_^+^‐capped surface. b) PLQYs of NH_4_^+^/C18A/C4. Inset shows optical photographs of the NH_4_^+^/C18A/C4 taken at different reaction times. c) The device architecture. d) The EQE of the devices with NPLs thickness of 4.5 and 7 nm. Reproduced with permission.^[^ ^75^ ^]^ Copyright 2022, American Chemical Society. e) A schematic diagram illustrating the CsPbBr_3_ NPLs treated with BTC. f) HR‐TEM images showing the structure of BTC‐treated CsPbBr_3_ NPLs. g) *EQE*–*J* curves of PeLEDs fabricated using both control and BTC‐treated NPLs, with an inset showing a photograph of an operational device with an active area of 2.0 × 1.5 cm. h) EL spectra of a device emitting at a constant wavelength of 465 nm under varying voltages, with the inset displaying the corresponding CIE color space plot. Reproduced with permission.^[^ ^178^ ^]^ Copyright 2024, American Chemical Society. i) Normalized UV absorption and PL emission spectra of CsPbBr_3_ NWs with diameters ranging from 2.5 to 32 nm. j) PLQY of CsPbBr_3_ NWs with different diameters. k) Time decay curves for NWs of varying diameters. Reproduced with permission.^[^ ^134^ ^]^ Copyright 2018, The Royal Society of Chemistry. l) PL emission spectra of films and EL spectra of CsCdBr_3_‐0.25 NR‐based LEDs, with insets showing the CIE coordinates of the EL emission and device photos under a 9 V bias. m) Current efficiency and EQE plotted against current density for the same LEDs. Reproduced with permission.^[^ ^183^ ^]^ Copyright 2012, Elsevier, Inc.

NPLs exhibit PL emission in deeper regions of the visible spectrum, making them ideal for applications requiring specific wavelengths, such as blue or UV light. However, despite their advantageous emission properties, NPLs typically display lower EQEs compared to QDs (Figure 6k). This reduced performance is due to factors such as low charge transport and optical anisotropy, as well as increased surface defects.^[^ ^178^, ^179^, ^180^ ^]^ With a larger surface area relative to their volume, NPLs are more prone to surface defects and trap states, which act as non‐radiative recombination centers, trapping charge carriers and reducing radiative efficiency. Additionally, the large surface‐to‐volume ratio intensifies the charge‐blocking effect of insulating ligands, further lowering the EQE. Chen et al.^[^ ^179^ ^]^ employed a strong sterically hindering ligand, 4‐dodecylbenzenesulfonic acid (DBSA), to synthesize amine‐free CsPbBr_3_ NPLs that achieve stable and pure‐blue light emission. By fine‐tuning the DBSA/Br ratio, they precisely controlled the steric hindrance and NPL thickness. Unlike traditional OA/OLA ligands, DBSA demonstrated stronger binding with the NPL surface, leading to slower ligand exchange and enhanced stability. The addition of alkali carbonates neutralized the precursors and passivated the surface, significantly boosting the PLQY. Wang et al.^[^ ^75^ ^]^ reported that NPLs capped with a short ligand, n‐butylamine (C4), and myristic acid (C14) and passivated with an ammonium bromide (NH_4_Br) additive exhibited high colloidal stabilities. Electrical performances and luminous efficiencies of these NPLs were significantly improved using the short conjugation ligand–phenethylammonium bromide post‐synthetic treatment. PLQYs of passivated NPLs were over 80% with narrow FWHM values of 16.7 nm at an emission wavelength of 463 nm. LED (ITO/PEDOT/PolyTPD/PeNCs/TPBi/LiF/Al) fabricated with these NPLs demonstrated a turn‐on voltage of 3.3 V, a luminance of 141 cd m^−^^2^, and EQE of 2%, which is the maximum efficiency reported for perovskite NPLs in the blue region (**Figure** **8**a–d).

Liu et al.^[^ ^181^ ^]^ passivated a perovskite NPL surface with 2,2‐(ethylenedioxy) bis(ethylammonium) sulfate (EDBESO_4_). EDBESO_4_‐treated CsPbBr_3_ NPLs exhibited considerably high PL quantum efficiencies and stabilities as compared to those of pristine CsPbBr_3_ NPLs capped with OAm and OA. PLQYs of EDBESO_4_‐treated CsPbBr_3_ NPLs were 85% with high color purities (FWHM, 13 nm) at an emission wavelength of 462 nm. Higher PLQYs originated from the interactions of EDBE^2+^ and SO_4_^2–^ with CsPbBr_3_ and filling of the Cs vacancies through binding of EDBE^2+^ to uncoordinated Pb atoms on the surface. Additionally, replacing the long capping ligands with shorter EDBESO_4_ enhances charge carrier transport, resulting in more efficient LEDs. EQEs of LEDs (ITO/PEDOT/PolyTPD/NPLs/TPBi/LiF/Al) were 1.77% with maximum luminances of 691 cd m^−2^. Wang et al.^[^ ^178^ ^]^ developed efficient and stable pure blue‐emitting CsPbBr_3_ NPLs by incorporating an aromatic ligand, 4‐bromothiophene‐2‐carboxaldehyde (BTC). The thiophene groups in BTC promote 2D growth while preventing out‐of‐plane ripening, enhancing the structural stability of the NPLs by interacting strongly with PbBr_6_^4−^ octahedra. The aromatic structure with delocalized π‐bonds improves charge transport, reduces band tail states, and suppresses Auger recombination. As a result, the PeLEDs with device structure ITO/PEDOT/PTAA/PEABr/PeNPLs/TPBi/LiF/Al exhibited efficient and stable blue emission at 465 nm, characterized by a narrow emission linewidth of 17 nm. This configuration achieved a maximum EQE of 5.4% and a luminance of 227 cd m^−2^, marking a significant advancement in the CsPbBr_3_ NPL LEDs (Figure 8e–h). **Table** **4** and Table S3 (Supporting Information) summarize the optoelectronic properties of perovskite NPLs and the performances of perovskite NPL‐based LEDs.

**Table 4:** Summary of the optoelectronic properties of perovskite NPLs and performances of perovskite NPLs‐based LEDs.

Composition of PeNCs	Thickness [nm]	Synthesis Method	Defect treatment	Device Structure	PL Peak Wavelength [nm]	PLQY [%]	FWHM [nm]	EQE [%]	PL Lifetime (ns)/^*^EL Lifetime [T_50_] [s]	Year	Reference
CsPbBr_3_	3.0	HI	No	‐	488	84.4	14	‐	‐	2015	[130]
CsPbBr_3_	2.5	HI	No	‐	449	54.61	‐	‐	4.33	2016	[192]
CsPbBr_3_	2.9	LARP	PbBr_2_	‐	475	67.0	‐	‐	4.2	2018	[127]
CsPbBr_3_	3.2	LARP	PbBr_2_	‐	486	67.7	‐	‐	4.1	2018	[127]
CsPbBr_3_	2.5	HI	No	‐	462	50	‐	‐	‐	2019	[193]
CsPbBr_3_	2.6	LARP	TOABr	‐	495		‐	‐	‐	2020	[194]
CsPbBr_3_	2.1	HI	OAmBr	‐	460	97.4	14.9	‐	5.89	2021	[195]
CsPbBr_3_:Ag^+^	2.6	LARP	Ag^+^‐doped	‐	460	98	14	‐	4.2	2021	[196]
CsPbBr_3_:Sb^3+^	3.0	LARP	Sb^3+^‐doped	‐	465	95	19	‐	1.48	2022	[197]
CsPbBr_3_:K^+^	2.5	LARP	K^+^‐doped	‐	450	87	‐	‐	8.85	2021	[198]
MAPb_0.3_Ce_0.7_Br_3_	1.6	Solvothermal	Ce^3+^‐doped	‐	454	100	24	‐	‐	2021	[199]
CsPbBr_3_:Zn^2+^	2.0	LARP	Zn^2+^‐doped	‐	461	90	15	‐	14.75	2022	[200]
CsPbBr_3_	1.2	HI	No	‐	430			‐	‐	2023	[201]
CsPbBr_3_	2.67	HI	ZnBr_2_	‐	454	87.2	16.8		16.69	2024	[202]
CsPbBr_3_	1.2	HI	OAmBr	‐	434	81.2	11	‐	10.13	2024	[203]
MAPbBr_3_	n = 5	LARP	No	ITO/PEDOT/PVK/NPLs/TPBi/LiF/Al	492	40‐90	24	0.23	27.2	2016	[78]
CsPbBr_3_	3.0	LARP	No	ITO/PEDOT/PolyTPD/ NPLs/TPBi/LiF/Al	463	96	12	0.124	3.96	2018	[204]
(Rb/Cs)PbBr_3_		HI	Rb^2+^‐doped	ITO/PEDOT/PolyTPD/ NPLs/TPBi/LIF/Al	464	60	18	0.11	6.73	2019	[205]
(Rb/Cs)PbBr_3_		HI	Rb^2+^‐doped	ITO/PEDOT/PolyTPD/ NPLs/TPBi/LIF/Al	490	90	24	0.69	5.61	2019	[205]
CsPbBr_3_		LARP	EDBESO_4_	ITO/PEDOT/PolyTPD/ NPLs/TPBi/LiF/Al	460	85	13	1.77	4.5/^*^1200	2022	[91]
CsPbBr_3_	2.0	LARP	PEABr	ITO/PEDOT/PolyTPD/ NPLs/TPBi/LiF/Al	463	81.6	16.7	2.0		2022	[75]
CsPbBr_3_:Zn^2+^	2.88	LARP	Zn^2+^‐doped	ITO/PEDOT/PVK/ NPLs/TPBi/LiF/Al	460	78	18	0.23		2023	[206]
CsPbBr_3_	2.0	LARP	BTC	ITO/PEDOT /PTAA/PEABr/PeNPLs/TPBi/LiF/Al	463	83	17	5.44	2.89/^*^860	2024	[178]
CsPbBr_3_	2.5	HI	P‐TPD	ITO/PEDOT/NPL‐polymer composite/TPBi/LiF/Al	495	72	‐	0.12	1.39	2024	[207]

TOABr = tetraoctylammonium bromide, P‐TPD = poly(4‐butyl‐*N,N*‐iphenylamine)

Compared to the QDs and NPLs, controlling the dimensions of NWs/NRs below the Bohr diameter through colloidal synthesis is challenging, primarily due to the difficulty in precisely managing their growth in one dimension at small scales. The strong confinement effect can lead to aggregation, resulting in color instability and broader FWHM of the emission spectrum. As a result, there are only a few studies on blue‐emitting perovskite NWs/NRs.^[^ ^140^ ^]^ Nevertheless, several research groups have reported NWs/NRs with high PLQY through controlled synthesis and defect passivation techniques. Imran et al. demonstrated the colloidal synthesis of CsPbBr_3_ perovskite NWs with tunable widths, ranging from the non‐confined regime to the strong quantum confinement regime. By introducing short aliphatic carboxylic acids like octanoic or hexanoic acid, they were able to achieve NWs with a PLQY as high as 77%. The PL emission could be tuned from green to blue based on the NW width. However, NWs with widths below ≈5 nm showed reduced stability, evidenced by additional PL and absorption peaks, along with a decline in PLQY. Liu et al.^[^ ^134^ ^]^ reported similar observations, further confirming this behavior in quantum‐confined NWs (Figure 8i–k).

Li et al.^[^ ^182^ ^]^ reported ultrathin CsPbBr_3_ perovskite NWs with diameters of 2.1 nm that demonstrated substantial PLQYs of ≈60% with FWHM of 27 nm at an emission wavelength of 447 nm. High PLQYs and excellent water resistances of these CsPbBr_3_ perovskite NWs can be attributed to surface passivation with thiourea. Guo et al.^[^ ^183^ ^]^ reported the synthesis of 1D CsCdBr_3_ NRs, where the [CdX_6_]^4–^ octahedra, sharing opposite faces, form continuous linear chains along the c‐axis. However, these NRs exhibit low PLQYs, limiting their potential for LED applications. To enhance their optoelectronic properties, PbBr_2_ was introduced as a dopant, resulting in Pb^2^⁺‐doped CsCdBr_3_ NRs. When the molar ratio of PbBr_2_ to CdBr_2_ was set at 0.25, the doped NRs achieved a PLQY of 48% with pure‐blue emission centered at 459 nm. This led to the successful fabrication of a pure‐blue LED with device structure ITO/PEDOT:PSS/NRs/TPBi/Liq/Al, achieving a maximum brightness of 225 cd m^−2^ and an EQE of 0.35%. The synthetic difficulties, alongside issues with charge transport, surface defects, and stability, contribute to this underexplored area in perovskite research (Figure 8l,m). **Table** **5** summarizes the optoelectronic properties of perovskite NWs/NRs and performances of perovskite NWs/NRs‐based LEDs.

**Table 5:** Summary of the optoelectronic properties of perovskite NWs/NRs and performances of perovskite NWs/NRs‐based LEDs.

Composition of PeNCs	Diameter [nm]	Synthesis Method	Defect treatment	Device Structure	PL Peak Wavelength [nm]	PLQY [%]	FWHM [nm]	EQE (%)	PL Lifetime [ns]	Year	Reference
CsPbBr_3_	3.4	HI	No		473	30	33		2.5	2016	[140]
CsPbBr_3_	4.1	HI	No		481	40	18		2.8	2016	[140]
CsPbBr_3_	5.1	HI	No		496	77	16		4.9	2016	[140]
CsPbBr_x_Cl_1‐x_	10	HI	PbBr_2_		466	30	‐		‐	2016	[141]
CsPbBr_3_	2.2	HI	No		465	30	25		‐	2016	[208]
CsPbBr_3_	2.5	One Pot	No		430	46	‐		4.35	2018	[134]
CsPbBr_3_	3.2	One Pot	No		450	51	‐		3.0	2018	[134]
CsPbBr_3_	3.5	HI	No		464	44	‐		8.1	2017	[209]
CsPbCl_3_	20	HI	Cu^2+^‐doped		420	‐	12		‐	2018	[210]
CsPbBr_3_	2.1	HI	No		447	60	27		‐	2019	[182]
CsPbBr_3_	3.8	Solvothermal	No		445	28.8	17		‐	2021	[211]
CsPbBr_3_	3.3	LARP	Mn^2+^‐doped		469	26.8	15		68.11	2022	[212]
CsCdBr_3_	15.9	HI	Pb^2+^‐doped	ITO/PEDOT:PSS/NRs/TPBi/Liq/Al	459	48	33	0.35	^*^870	2022	[183]

## 9 Challenges of PeNC‐based Blue LEDs

**Figure 9:**
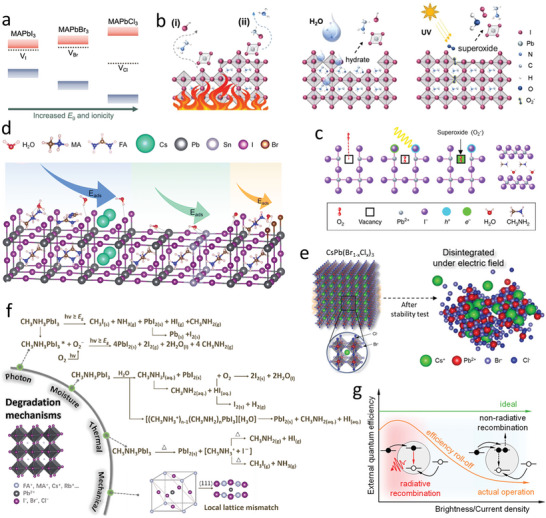
a) The energy of the (0/+) transition level of halide vacancies in CH_3_NH_3_PbX_3_ perovskites refers to the energy at which a neutral halide vacancy becomes positively charged by losing an electron. Reproduced with permission.^[^ ^26^ ^]^ Copyright 2017, The Authors, published by Springer Nature. b) Graphical depictions of the deterioration of perovskite crystals caused by heat, moisture, exposure to UV radiation, and oxygen. Reproduced with permission.^[^ ^118^ ^]^ Copyright 2022, Wiley‐VCH. c) Reaction steps of O_2_ with CH_3_NH_3_PbI_3_, showing oxygen diffusion and penetration into the lattice and its transformation to other compounds. Reproduced with permission.^[^ ^227^ ^]^ Copyright 2017, The Authors, published by Springer Nature. d) Water adsorption on the different functional groups terminated perovskite surface. Reproduced with permission.^[^ ^234^ ^]^ Copyright 2024, The Royal Society of Chemistry and the Chinese Chemical Society. e) Schematic representation of the structural deformation of the perovskite under operational stress. Reproduced with permission.^[^ ^66^ ^]^ Copyright 2024, The Authors, published by Wiley‐VCH. f) Mechanism of perovskite degradation under both extrinsic and intrinsic stresses. Reproduced with permission.^[^ ^223^ ^]^ Copyright 2022, Elsevier, Inc. g) Schematic representation of efficiency roll‐off of the operating LED. Reproduced with permission.^[^ ^242^ ^]^ Copyright 2023, American Chemical Society.

In the rapidly evolving domain of LEDs, blue PeLEDs have emerged as a focal point of research due to their potential to exhibit high efficiencies and color purities. Despite significant advancements in PeLED technology, realizing PeLEDs that emit in the blue region of the spectrum remains particularly challenging in terms of both efficiency and stability for commercialization in display applications. Achieving high efficiency in blue PeLEDs is hampered by numerous challenges stemming from material properties, recombination dynamics, and device‐level inefficiencies. One of the primary issues with PeNCs, especially those emitting in the blue spectrum, is their increased surface deep‐level defects arising from the wider band gap.^[^ ^26^ ^]^ Surface ligands, which stabilize the PeNCs, play a crucial role in preventing such defects. However, ligand instability is a common problem, particularly in blue‐emitting PeNCs, where excessive doping with chloride ions or other passivating agents can strip ligands from the PeNC surface, further creating deep‐level defects.^[^ ^27^ ^]^ In chloride perovskites, a higher defect density and deep‐level defects are often introduced due to chloride vacancies. These vacancies arise from the small size and lightweight nature of Cl⁻ ions and their deep (0/+) transition state, which refers to the energy at which a neutral halide vacancy becomes positively charged by losing an electron (**Figure** **9**a).^[^ ^26^, ^150^, ^151^ ^]^ These defects act as non‐radiative recombination centers, trapping charge carriers and preventing radiative recombination, which lowers the PLQY and overall efficiency of the device.

When PLQY measurements are conducted under controlled conditions, especially in the solution state, fewer defects are generated, resulting in improved PL efficiency. However, surface defects and trap states become more pronounced in the device conditions, resulting in non‐radiative recombination that reduces EL efficiency. Efficient charge injection and balanced transport of electrons and holes are essential for high‐efficiency LEDs. Due to the inherent wider band gap characteristic of blue‐emitting perovskites, energy level mismatches between the perovskite layer and the charge transport layers in the blue PeLEDs contribute to inefficient charge injection.^[^ ^28^, ^213^, ^214^, ^215^ ^]^ Additionally, high defect density, deep‐level defects, or strong quantum confinement in blue perovskites typically result in poorer charge transport properties. This inadequate charge injection and transport within the device lead to an imbalance in electron‐hole recombination within the EML, causing some carriers to recombine non‐radiatively, which reduces the overall EQE of the device, despite the high PLQY of the materials under optical measurement conditions.^[^ ^216^, ^217^, ^218^ ^]^

The practical implementation of PeLEDs is significantly hindered by both the external and internal instabilities of PeNCs as well as the operational instability of the device.^[^ ^219^, ^220^ ^]^ Extrinsic instabilities of PeNCs originate from environmental factors such as O_2_, moisture (H_2_O), light, and thermal and mechanical stresses, which can trigger phase transformations to non‐perovskite structures during fabrication and operation (Figure 9b).^[^ ^221^, ^222^, ^223^ ^]^ MAPbX_3_ perovskites are more vulnerable, and exposure to O_2_ can trigger oxidative stress in the perovskite structure, further compromising the perovskite stability and efficiency.^[^ ^224^, ^225^, ^226^ ^]^ Exposure to O_2_ leads to the degradation of perovskite materials due to the formation of superoxide species, which leads to the degradation and damage of contacting layers, impacting the performances of PeLEDs.^[^ ^118^, ^227^ ^]^ Figure 9c shows O_2_ diffusion and penetration into the MAPbI_3_ perovskite lattice and transformation of MAPbI_3_ into other compounds. Oxidation and phase transformation are favored by light exposure and bias current. According to density functional theory (DFT) calculations, in the absence of photogenerated or biased electrons, oxidation is unfavorable due to the positive enthalpy change. However, in the presence of light or biased current, O_2_ acts as an electron scavenger, which then reacts with the excited perovskite molecules.^[^ ^227^ ^]^ Negative enthalpy change indicates the thermodynamic favorability of the reaction. Anion vacancies act as defect sites, initiating the O_2_‐induced degradation of the perovskite structure and formation of PbI_2_, I_2_, H_2_O, and CH_3_NH_2_. Generated water further degrades the perovskite structure by dissolving the organic cation and opening the PbI_2_ surface for thermodynamically stable iodate formation.^[^ ^221^, ^228^ ^]^

Interaction of water molecules with the perovskite crystal structure also leads to the formation of hydrates and consequent breakage of bonds in the crystal lattice or phase transformation.^[^ ^222^, ^229^, ^230^, ^231^, ^232^ ^]^ MAPbX_3_ perovskites initially form monohydrate or dihydrate intermediates, which are unstable and later decompose into other products. Water molecules interact with the perovskite structure through H bonding with the ‐NH_3_ functional group of the MAX‐terminated surface or the iodide functional group of the AX‐terminated surface. Contrarily, the PbX_2_‐terminated surface can form an H bond with the ‐CH_3_ functional group or O atom of the water molecules through its anion vacancy sites (Figure 9d).^[^ ^233^, ^234^ ^]^ FAPbX_3_ and CsPbX_3_ perovskites are more stable than MAPbX_3_ and typically transform into other phases, where water molecules act as catalysts and trigger the transformation.^[^ ^222^, ^232^ ^]^

Moreover, continuous exposure of blue perovskites to high‐energy photons can result in the breakdown of chemical bonds and the formation of defects in the crystal lattice.^[^ ^235^, ^236^ ^]^ Another factor contributing to the low stabilities of blue‐emitting perovskites is thermal instability resulting from Joule heating at the higher operating energies required for blue emission. Electrochemical reactions also induce alterations in surface compositions and oxidation states at the interfaces of perovskite layers, subsequently reducing the energy barrier at the exposed contacts. These reactions also serve as sources for the diffusion of defect states.^[^ ^237^ ^]^ Intrinsic ionic natures of perovskite materials and susceptibilities of these materials to degradation under operational conditions can lead to ion migration under electrical stress, resulting in a shift in the emission wavelength and spectral instability over time. High vulnerabilities of blue‐emitting perovskites, particularly those with mixed‐halide compositions, to degradation is a significant concern for their application in blue PeLEDs. Figure 9e shows a schematic of the structural deformation of a perovskite under operational stress, whereas Figure 9f depicts the degradation mechanisms of the perovskite under both extrinsic and intrinsic stresses.

Efficiency roll‐offs in blue PeLEDs are a formidable obstacle in the pursuit of high‐brightness applications. These roll‐offs at high intensities may result from luminescence quenching or an excessive number of charge carriers passing through the device without forming electron‐hole pairs.^[^ ^238^ ^]^ Intricate natures of efficiency roll‐offs in PeLEDs stem from various underlying mechanisms, prominently involving charge imbalance and non‐radiative recombination processes.^[^ ^239^, ^240^, ^241^ ^]^ Accumulation of excess charge carriers elevates the possibility of non‐radiative recombination events, further exacerbating efficiency roll‐off. These non‐radiative processes, occurring when electrons and holes recombine without emitting photons, are exacerbated by defects in the perovskite crystal structure. Figure 9g shows efficiency roll‐offs by depicting the variation of the EQE curve with an increase in brightness or current density in an operating LED under ideal and actual conditions. Graph schemes demonstrate the radiative and non‐radiative recombination pathways generated in a perovskite QD when charge carriers are injected into this QD.

## 10 Approaches to Resolve Issues of PeNC‐based Blue LEDs

Enhancing both the performances and stabilities of PeNC‐based blue LEDs is complicated owing to the intrinsic properties of perovskites and the demanding requirements of LED applications. Researchers have developed various strategies to tackle these issues, each offering unique insights into the interplay between material properties and device performance. These strategies represent a convergence of innovations in material engineering,^[^ ^29^, ^74^, ^91^, ^243^, ^244^, ^245^, ^246^, ^247^ ^]^ device architecture,^[^ ^248^, ^249^, ^250^ ^]^ and encapsulation techniques,^[^ ^21^, ^251^ ^]^ reflecting the adaptive and problem‐solving ethos of this field.

**Figure 10:**
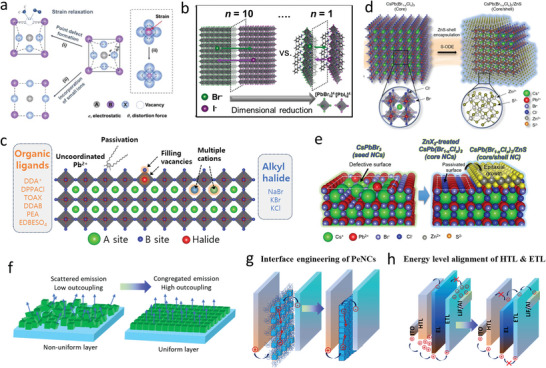
Schematic overview of approaches for achieving highly stable and efficient blue PeLED: a) Stability enhancement of the perovskite crystal through doping or point defect formation. Reproduced with permission.^[^ ^118^ ^]^ Copyright 2022, Wiley‐VCH. b) Dimension reduction of perovskite crystal to reduce ion segregation. Reproduced with permission.^[^ ^88^ ^]^ Copyright 2020, American Chemical Society. c) Defect passivation with different organic and inorganic ligands and salts. d,e) Epitaxial core–shell perovskite architecture for enhancing stability. Reproduced with permission.^[^ ^21^ ^]^ Copyright 2022, The Authors, published by Wiley‐VCH. f) Perovskite layer engineering to enhance outcoupling. g,h) Energy level alignment of the HTL and ETL with high charge transfer mobility.

Material engineering is pivotal for enhancing the efficiency and stability of perovskite materials used in various optoelectronic devices including PeLEDs.^[^ ^29^, ^74^, ^91^, ^243^, ^244^, ^245^, ^246^, ^247^ ^]^ Selecting appropriate compositions for PeNCs is crucial for the stabilities of PeNCs. PeNCs with mixed‐cation or mixed‐halide compositions exhibit high stabilities due to the reduction of phase segregation and susceptibility to environmental factors such as moisture and temperature.^[^ ^83^, ^118^, ^252^, ^253^ ^]^ Ion segregation can also be mitigated by reducing the dimensionalities of perovskite crystals.^[^ ^88^ ^]^ Furthermore, tailoring the crystal structures of PeNCs through doping, alloying, or lattice strain can enhance the stabilities and optoelectronic properties of PeNCs.^[^ ^254^ ^]^ Engineering the crystal lattice can mitigate defects and improve charge carrier mobility, leading to more efficient devices (**Figure** **10**a,b).^[^ ^255^, ^256^, ^257^ ^]^

Surface passivation has emerged as a pivotal technique for addressing the abovementioned challenges, effectively mitigating surface defects that lead to radiative recombination and subsequent material degradation by obstructing the migration pathways of these defects. Extensive research has identified a range of point defects, notably uncoordinated surface cations/anions, and corresponding vacancies, as the principal contributors to ion migration and inferior spectral stability. Surface defects are predominantly associated with uncoordinated Pb^2+^ and X^−^. Numerous passivation agents or ligands, including those with alkali and transition metal halides,^[^ ^74^, ^258^ ^]^ organic halide molecules,^[^ ^259^, ^260^, ^261^ ^]^ and specifically charged organic molecules,^[^ ^91^, ^160^, ^258^ ^]^ have demonstrated efficacies in rectifying these defects, thereby enhancing the efficiency and stability of PeNCs. (Figure 10c).

Encapsulation of core–shell structures represents one of the most sophisticated and practical approaches to the advancement of PeLEDs and offers a transformative solution to several critical challenges in the PeLED technology, which has been successfully implemented in semiconductor QDs.^[^ ^262^ ^]^ Although few attempts have been made in this regard, some have successfully created core–shell perovskite structures (namely, epitaxial and nonepitaxial structures) that have improved device performance and led to unprecedented improvements in PLQY, material stability, and lifetime extension.^[^ ^21^, ^263^, ^264^, ^265^, ^266^ ^]^ Although growing an epitaxial shell on PeNCs is challenging, growing an epitaxial semiconductor with a wide band gap on PeNCs is highly attractive due to the ability of this semiconductor shell to further passivate defects and enhance charge carrier confinement in PeNCs, leading to enhanced radiative recombination and luminescence (Figure 10d,e).^[^ ^21^, ^22^, ^267^, ^268^ ^]^

Device engineering, specifically focusing on uniform layer engineering, interface engineering, and band gap alignments of HTLs and ETLs, is crucial for enhancing charge injection and transport. These techniques help reduce energy losses and improve the overall device performance.^[^ ^83^, ^148^, ^269^ ^]^ Uniformity of the perovskite layer minimizes non‐radiative recombination, ensuring consistent light emission across the PeLED surface by maximizing the luminescence efficiency and color uniformity. Kim et al.^[^ ^270^ ^]^ fabricated a uniform layer by spin coating a dilute solution of PeNCs multiple times and achieved high device performance using this layer. Furthermore, uniformity of the perovskite layer reduces the defect density and ensures consistent device performance over time, thereby enhancing the stabilities and reliabilities of PeLEDs.^[^ ^148^, ^271^ ^]^ A uniform layer with specific orientations of PeNCs, specifically NPLs, increases the outcoupling efficiency, resulting in high PeLED performance. Cui et al.^[^ ^180^ ^]^ demonstrated efficient electroluminescence from an in situ‐grown perovskite film consisting of a monolayer of face‐on oriented NPLs. With ≈84% of the perovskite NPLs featuring horizontal TDMs, the film achieves a light‐outcoupling efficiency of ≈31%, significantly surpassing the efficiencies of isotropic emitters.

Interface engineering and energy‐level alignments of HTL and ETL in PeLEDs are essential for optimizing charge injection, reducing non‐radiative recombination, preventing PeLED degradation, and enhancing light extraction efficiency.^[^ ^28^, ^118^, ^272^, ^273^ ^]^ Yuan et al.^[^ ^28^ ^]^ developed a high‐mobility ETM to improve the carrier injection balance in blue PeLEDs. Tailored asymmetric anthracenyl structure achieves an electron mobility of 2.7 × 10⁻⁴ cm^2^·V⁻¹·s⁻¹, nearly 20 times that of commonly used ETM‐TPBi (1.1 × 10⁻⁵ cm^2^·V⁻¹·s⁻¹). Consequently, the corresponding PeLEDs demonstrate significantly higher EQEs of 13.2% and lower turn‐on voltages of 2.2 V, outperforming TPBi‐based devices, which exhibit EQEs of 8.3% and turn‐on voltages of 3.2 V. Long‐chain ligands of PeNCs hinder charge carrier transport across interfaces. Replacing these long‐chain ligands with shorter‐chain or inorganic ligands improves charge transport through the interfaces, thereby increasing EQE.^[^ ^89^, ^90^ ^]^ Interface engineering also minimizes efficiency roll‐offs in high‐brightness PeLEDs by reducing Auger recombination.^[^ ^274^, ^275^ ^]^ Yao et al.^[^ ^275^ ^]^ developed an innovative QD film structure for PeLEDs by inserting a ZnCl_2_ barrier layer into pure‐blue CsPbBr_3_ QDs. This ultrathin interlayer localized the charge recombination region in a sandwich panel, reducing exciton quenching and Auger recombination. This approach balanced electron and hole transport, leading to a stable, high‐purity blue emission at 469 nm with an EQE of 5.0%. PeLEDs maintained over 90% of initial EQEs at 10 000 cd m^−^^2^ and achieved maximum luminances of 10410 cd m^−^^2^. They also exhibited considerable durabilities, with half‐lifetimes of 59 h at 100 cd m^−^^2^, setting a record for blue and cyan PeLEDs.

These efforts have collectively improved the device stability, and efficiency, rendering interface engineering and energy‐level alignment key focuses on PeLED research and development (Figure 10f–h).

## 11 Conclusion

This review extensively explores the advancements and challenges in colloidal PeNC‐based blue LEDs, highlighting the promise of these LEDs for next‐generation displays. Tunable compositions and size confinements of PeNCs via colloidal methods, specifically ion exchange with anions, facilitate the synthesis of blue‐emitting PeNCs. Quantum confinement effects lead to band gap enlargement and blue shift in the PL emission with a decrease in the PeNC size to the Bohr radius. Recent developments in blue PeNCs have achieved near‐unity PLQYs, exhibiting high color purities with narrow FWHM values of 12 nm, while PeNC‐based LEDs have achieved EQEs of 21.3 and 26.4% at the wavelength of 470 and 480 nm, respectively. However, these blue LEDs still face significant challenges in efficiency and stability as compared to the cases of red and green LEDs. Achieving high efficiency in blue PeLEDs is challenging due to surface defects, non‐radiative recombination, and device inefficiencies. The wider band gap of blue‐emitting PeNCs makes them prone to chloride vacancies, trapping charge carriers and lowering PLQY. Ligand instability worsens defect formation. Additionally, poor charge injection and transport, along with energy level mismatches between perovskite and charge transport layers, cause imbalanced recombination, reducing EL efficiency. Environmental factors such as O_2_, moisture, light, and thermal and mechanical stresses induce extrinsic instability, leading to phase transformations to non‐perovskite structures. O_2_ degrades perovskite materials through superoxide formation, whereas water causes hydration and bond breakage. High‐energy photons and Joule heating result in chemical bond breakdown, defect formation, and ion migration, causing spectral instability and efficiency roll‐off. These issues hinder the practical implementation of PeNC‐based blue LEDs, necessitating further research and development to overcome these obstacles. Multidisciplinary strategies have been adopted to address the limitations of PeNC‐based blue LEDs. Techniques including A‐site and B‐site doping and halide‐based surface passivation help rectify defects. Core–shell structures and all‐inorganic architectures improve the efficiencies and stabilities of PeNCs by mitigating ion migration. A uniform layer with specifically oriented PeNCs, particularly NPLs, boosts the outcoupling efficiency, thereby improving the overall PeLED performance. However, challenges persist regarding perovskite layer degradation. Innovations in HTLs, such as binary‐blend HTLs, and ETLs, for example, modified ZnO ETLs, are essential for better charge injection and transport. Interface engineering, including the modification of PEDOT layers, also contributes to high stability and performance. Through systematic reviews and analyses of current research trends, this review aims to highlight the path forward for the future development of PeNC‐based blue LEDs.

## Conflict of Interest

The authors declare no conflict of interest.

## Supporting information



Supporting Information
