# Supplementary material for: Colloidal Perovskite Nanocrystals for Blue‐Light‐Emitting Diodes and Displays
Source: Adv Sci (Weinh). 2025 Mar 9;12(15):2409736. doi: 10.1002/advs.202409736 (PMC12005814; doi:10.1002/advs.202409736)
Supplement: Supplementary file 1 — Supporting Information [file ADVS-12-2409736-s001.pdf]

ADVANCED SCIENCE
Open Access

Supporting Information

for *Adv. Sci.*, DOI 10.1002/advs.202409736

Colloidal Perovskite Nanocrystals for Blue-Light-Emitting Diodes and Displays

*Md Aftabuzzaman, Yongju Hong, Sangyeon Jeong, Ratiani Levan, Seung Jin Lee, Dong Hoon Choi and Kwangyeol Lee**

WILEY-VCH

Supporting Information

Colloidal Perovskite Nanocrystals for Blue-Light-Emitting Diodes and Displays

*Md Aftabuzzaman, Yongju Hong, Sangyeon Jeong, Ratiani Levan, Seung Jin Lee, Dong Hoon Choi, Kwangyeol Lee**

Table S1. Summary of the optoelectronic properties of perovskite nanocubes and performances of perovskite nanocube-based LEDs.

Composition of Nanocubes	Synthesis Method	Defect treatment	Device Structure	PL Wavelength (nm)	PLQY (%)	FWHM (nm)	EQE (%)	Year	Ref
$CsPbCl_3$	LARP	No		405	10	12		2016	[1]
$CsPbCl_{1.5}Br_{1.5}$	LARP	No		455	37	16		2016	[1]
$CsPbClBr_2$	LARP	No		478	70	18		2016	[1]
$CsPbBr_3$:Al^{3+}	HI	Al^{3+}-doped		456	42	16		2017	[2]
$CsPb_{1-x}Sn_xBr_3$	HI	Sn^{2+}-doped		479	~ 60	80		2017	[3]
$CsPb_{1-x}Sn_xBr_3$	HI	Sn^{2+}-doped		484	~ 60	80		2017	[3]
$CsPb_{1-x}Cd_xBr_3$	HI	Cd^{2+}-doped		452	~ 60	80		2017	[3]
$CsPb_{1-x}Cd_xBr_3$	HI	Cd^{2+}-doped		483	~ 60	80		2017	[3]
$CsPb_{1-x}Zn_xBr_3$	HI	Zn^{2+}-doped		462	~ 60	80		2017	[3]
$CsPbCl_3$-Mn^{2+}/K^+	HI	Mn^{2+}/K^+ doped		480	67	12		2017	[4]
$CsPbCl_3$:Y	HI	Y^{3+}-doped		404	60			2018	[5]
(Rb/Cs)PbCl	HI	Rb^+-doped		406	13			2018	[6]
$CsPbBr_xCl_{3-x}$	HI	No		467	23	14		2018	[7]
$CsPbCl_3$	HI	$NaBF_4$ or NH_4BF_4		402	50	14		2018	[8]
$CsPbCl_3$	HI	No		408	65	11		2018	[9]
$MAPbCl_3$	HI	No		404	5	15		2018	[9]
$FAPbCl_3$	HI	No		407	2	16		2018	[9]

$CsPbBr_xCl_{3-x}$:Pb^{2+}	HI	Pb^{2+}-doped		463	29			2018	[10]
$CsPbBr_xCl_{3-x}$:Zn^{2+}	HI	Zn^{2+}-doped		463	50			2018	[10]
$CsPbBr_xCl_{3-x}$:Cd^{2+}	HI	Cd^{2+}-doped		464	57			2018	[10]
$CsPbCl_3$:F^+/La^{3+}	HI	F^+/La^{3+}-doped		411	36			2019	[11]
$CsPbCl_3$:Cu	HI	Cu^{2+}-doped		403	12	12.3		2019	[12]
$CsPbBr_xCl_{3-x}$:Ni^{2+}	LARP	Ni^{2+}-doped		432	73			2020	[13]
$CsPbBr_xCl_{3-x}$:La^{3+}	HI	La^{3+}-doped		448	50.4	22.8		2020	[14]
$CsPbBr_xCl_{3-x}$:La^{3+}	HI	La^{3+}-doped		451	57.8	20.6		2020	[14]
$CsPbBr_xCl_{3-x}$:La^{3+}	HI	La^{3+}-doped		456	66.8	22.6		2020	[14]
$FAPbBr_3$	HI	No		476	45			2021	[15]
$CsPb(Cl/Br)_3$	HI	No	ITO/PEDOT/PVK/PeNCs/TPBi/LiF/AI	452	60	23	0.07	2015	[16]
$CsPbBr_{1.5}Cl_{1.5}$	HI	No	ITO/ZnO/PeNCs/TFB/MoO_3/Al	480	45	17	0.0074	2016	[17]
$CsPbBr_xCl_{3-x}$	HI	No	ITO/NiOx/PeNCs/TPBi/LiF/Al	470	-	20	0.07	2017	[18]
$CsPbBr_xCl_{3-x}$	HI	No	ITO/PEDOT/TFB/PFI/PeNCs/TPBi/LiF/Al	469	9	23	0.5	2018	[19]
$CsPbBr_xCl_{3-x}$	HI	No	PEDOT/TFB/PFI/PeNCs/TPBi/LiF	481			0.44	2018	[19]
$CsMn_yPb_{1-y}BrxCl_{3-x}$	HI	Mn^{2+}-doped	ITO/PEDOT/TFB/PFI/PeNCs/TPBi/LiF/Al	466	28	17.9	2.12	2018	[20]
$CsPb(Br/Cl)_3$	HI	No	ITO/PEDOT/Poly-TPD/CBP/PeNCs/B3PYMPM/LiF/Al	463	37	13.6	1.2	2019	[21]
Ni^{2+}-$CsPbCl_{1.7}Br_{1.3}$	HI	$SOCl_2$ and $SOBr_2$	ITO/PEDOT/Poly-TPD/PeNCs/TPBi/LiF/Al	460	58	14.6	1.35	2019	[22]
$CsPbBr_xCl_{3-x}$	HI	DDAB and DDAC	ITO/PEDOT/Poly-TPD/PeNCs/B3PYMPM/TPBi/LiF/Al	487	48	20	3.5	2019	[23]

$CsPbBr_xCl_{3-x}$	HI	DDAB and DDAC	ITO/PEDOT/Poly-TPD/PeNCs/B3PYMPM/TPBi/LiF/Al	476	32	19	2.25	2019	[23]
$CsPbBr_xCl_{3-x}$	HI	DDAB and DDAC	ITO/PEDOT/Poly-TPD/PeNCs/B3PYMPM/TPBi/LiF/Al	462	25	19	2.25	2019	[23]
$(Rb_{0.33}CS_{0.67})_{0.42}FA_{0.58}PbCl_{1.25}Br_{1.75}$	HI	No	ITO/Poly-TPD/PeNCs/TPBi/Ba/Al	476	49.8	20	0.61	2019	[24]
$CsPbBr_xCl_{3-x}$	HI	DDAB and DDAC	ITO/PEDOT/Poly-TPD/PeNCs/TPBi/LiF/AI	479	60	19	0.86	2019	[25]
$CsPbBr_xCl_{3-x}$	HI	DDAB and DDAC	ITO/PEDOT/Poly-TPD/PeNCs/TPBi/LiF/Al	467	50	18	0.44	2019	[25]
$CsPbBr_xCl_{3-x}$	HI	DDAB and DDAC	ITO/PEDOT/Poly-TPD/PeNCs/TPBi/LiF/Al	485	70	18	0.86	2019	[25]
$Cs_xRb_{1-x}PbBr_3$	LARP	Rb-doped	ITO/PEDOT/PeNCs/TmPyPB/LiF/Al	492	25	20	0.17	2019	[26]
$CsPbBr_xCl_{3-x}$	LARP	SDSA	ITO/PEDOT/Poly-TPD/PeNCs/TPBi/Ca/Ag	496	42	18	2.6	2020	[27]
$CsPbBr_xCl_{3-x}:Nd^{3+}$	HI	Nd^{3+}-doped	ITO/PEDOT/TFB/PeNCs/TPBi/LiF/Al	478	55	15	2.7	2020	[28]
$(K/Cs)PbBr_xCl_{3-x}$	LARP	K^{+}-doped	ITO/PEDOT/Poly-TPD/PeNCs/TPBi/LiF/Al	477	27	20	1.96	2020	[29]
$(Cs/FA)PbBr_xCl_{3-x}:Cu$	LARP	Cu^{2+}-doped	ITO/PEDOT:PSS/PTAA/PeNCs/TOPO/TPBi/LiF/Al	490	67	19	5.02	2020	[30]
$CsPbBr_xCl_{3-x}$	HI	ADDA	ITO/PEDOT/TFB/PeNCs/TPBi/LiF/Al	456	31	15	0.49	2020	[31]
PEA-$CsPb(Cl_x/Br_{1-x})_3$	HI	PEACl	ITO/PEDOT/PVK/PeNCs/TmPyPB/LiF/Al	462	70.2	19	0.77	2020	[32]
$CsPbBr_{3-x}Cl_x$	LARP	DDAB and DDAC	ITO/PEI/ZnO/PeNCs/PVK/V_2O_5/Al	454	33.08	16.5	0.02	2021	[33]
$CsPbBr_{3-x}Cl_x$	LARP	DDAB and DDAC	ITO/PEI/ZnO/PeNCs/PVK/V_2O_5/Al	465	46.92	16.4	0.027	2021	[33]
$CsPbBr_{3-x}Cl_x$	LARP	DDAB and DDAC	ITO/PEI/ZnO/PeNCs/PVK/V_2O_5/Al	477	60.37	15.7	0.04	2021	[33]
$CsPbBr_{3-x}Cl_x$	LARP	DDAB and DDAC	ITO/PEI/ZnO/PeNCs/PVK/V_2O_5/Al	480	60.92	15.4	0.045	2021	[33]
$CsPbBr_{3-x}Cl_x$	LARP	DDAB and DDAC	ITO/PEI/ZnO/PeNCs/PVK/V_2O_5/Al	488	70	13.2	0.053	2021	[33]

$CsPb(Cl/Br)_3$	HI	$GeBr_3$	ITO/PEDOT/PTAA/PeNCs/TPBi/ LiF/Al	463	90	13	0.05	2022	[34]

TMOS = tetramethyl orthosilicate, SDSA = sodium dodecylbenzenesulfonate, ADDA = Adamantane-1,3-diamine, PEACl = Phenethylammonium chloride, DAT = n-dodecyl ammonium thiocyanate, DDAC = di-n-decyl dimethylammonium chloride, DDABr = di-n-decyl dimethylammonium bromide.

Table S2 Summary of the optoelectronic properties of perovskite QDs and performances of perovskite QDs-based LEDs.

Composition of PeNCs	Size (nm)	Synthesis Method	Defect treatment	PL Peak Wavelength (nm)	PLQY (%)	FWHM (nm)	Year	Ref
$MAPbCl_3$	3.3	LARP	No	407	50	34	2015	[35]
$MAPbCl_{2.1}Br_{0.9}$	3.3	LARP	No	446		17	2015	[35]
$MAPbCl_{0.6}Br_{2.4}$	3.3	LARP	No	467		32	2015	[35]
$MAPbBr_3$	1.5	LARP	No	403	5		2016	[36]
$CsPbBr_3$	3.1	LARP	No	457	40.3		2016	[37]
$CsPbBr_3$	3.5	LARP	No	465	51.7		2016	[37]
$CsPbBr_3$	2.4	HI	No	453	50	22	2016	[38]
$CsPbBr_3$	3.2	HI	No	467	38	33	2016	[39]
$CsPbBr_3$	2.6	LARP	No	456	27		2016	[40]

Table S3 Summary of the optoelectronic properties of perovskite NPLs and performances of perovskite NPLs-based LEDs.

Composition of PeNCs	Thickness (nm)	Synthesis Method	Defect treatment	Device Structure	PL Peak Wavelength (nm)	PLQY (%)	FWHM (nm)	EQE (%)	Year	Ref
$MAPbBr_3$	3	LARP	No		469	12.47			2015	[41]
$MAPbBr_3$	2	LARP	No		454	2.81			2015	[41]
$MAPbBr_3$	1	LARP	No		427	0.43			2015	[41]
$CsPbBr_3$	n = 3	HI	No		462	2.6			2015	[42]
$CsPbBr_3$	n = 4	HI	No		477	44.7			2015	[42]
$CsPbBr_3$	3.0	HI	No		459	31	19		2016	[43]
$MAPbBr_3$	2.0	LARP	No		488	50			2017	[44]
$CsPbBr_3$	1.2	LAPR	$PbBr_2$		433	49.4			2018	[45]
$(RNH_3)_2(CH_3NH_3)_{n-1}Pb_nBr_{3n+1}$		LARP	No		403	13.8	11		2016	[46]
$(RNH_3)_2(CH_3NH_3)_{n-1}Pb_nBr_{3n+1}$		LARP	No		442	24.5	16		2016	[46]
$(RNH_3)_2(CH_3NH_3)_{n-1}Pb_nBr_{3n+1}$		LARP	No		461	25.8	16		2016	[46]
$MAPbBr_3$		LARP	No		474	2.5			2016	[47]
$CsPbBr_3$	3.0	LARP	No		458	25	23		2017	[48]
$MAPbBr_3$	1.5	LARP	No		446	33			2017	[49]
$MAPbBr_3$	3.3	LARP	No		480	28			2017	[49]
$MAPbBr_3$	1.6	LARP	No		447	32			2017	[44]
$CsPbBr_3$	2.4	LARP	HPA		450	40	15		2020	[50]
$CsPbBr_3$	2.0	LARP	No		455	38	18		2020	[51]
$FAPbBr_3$	2.0	LARP	DDAB		440	46			2020	[52]
$CsPbBr_3$	1.9	HI	No		455	5			2021	[15]

$FAPbBr_3$	1.7	HI	No		435	25			2021	[15]
$MAPbBr_3$	n = 3	LARP	No	ITO/PEDOT/PVK/NPLs/TPBi/LiF/Al	456	40-90	18	0.024	2016	[53]
$CsPbBr_3$	2.0	LAPR	$PbBr_2$	ITO/PEDOT/PolyTPD/NPLs/TPBi/Ca/Ag	457	60.3	20	0.057	2018	[45]
$CsPbBr_3$	3.1	HI	No	ITO/PEDOT/PolyTPD/NPLs/TPBi/LiF/Al	480			0.1	2018	[54]
$CsPbBr_3$	3.41	LARP	DDAB	ITO/PEDOT/PolyTPD/NPLs/TPBi/LiF/Al	469	12		1.42	2019	[55]
PEA_2PbBr_4	4.0	LARP	No	ITO/PEDOT/PVK/TAPC/NPLs/TPBi/Ca/Ag	410	12-25	11.6	0.31	2019	[56]
$CsPbBr_3$	1.7	LARP	$PbBr_2$	ITO/PEDOT/PolyTPD/TFB/NPLs/TPBi/Ca/Ag	460	14	16	0.3	2019	[57]
$CsPbBr_3$	3.1	LARP	$PbBr_2$	ITO/PEDOT/PolyTPD/TFB/NPLs/TPBi/Ca/Ag	487	17	25	0.55	2019	[57]
$FAPbBr_3$	1.4	LARP	OAmBr	ITO/PEDOT/PolyTPD/NPLs/TmPyPB/LiF/Al	439	50	17	0.14	2020	[58]
$CsPbBr_3$		LARP	PEI	ITO/PEDOT/PTAA/NPLs/TPBi/LiF/Al	457	40	19.5	0.8	2021	[59]

PEI = polyethyleneimine, DDAB = di-n-decyl dimethylammonium bromide, [2]dimethyl-dioctadecyl-ammonium bromide, HPA = hexylphosphonate,

References

[1] X. Li, Y. Wu, S. Zhang, B. Cai, Y. Gu, J. Song, H. Zeng, *Adv. Funct. Mater.* **2016**, *26*, 2435.

[2] M. Liu, G. Zhong, Y. Yin, J. Miao, K. Li, C. Wang, X. Xu, C. Shen, H. Meng, *Adv. Sci.* **2017**, *4*, 1700335.

[3] W. Van der Stam, J. J. Geuchies, T. Altantzis, K. H. W. Van Den Bos, J. D. Meeldijk, S. Van Aert, S. Bals, D. Vanmaekelbergh, C. De Mello Donega, *J. Am. Chem. Soc.* **2017**, *139*, 4087.

[4] W. Xu, F. Li, F. Lin, Y. Chen, Z. Cai, Y. Wang, X. Chen, *Adv. Opt. Mater.* **2017**, *5*, 1700520.

[5] G. H. Ahmed, J. K. El-Demellawi, J. Yin, J. Pan, D. B. Velusamy, M. N. Hedhili, E. Alarousu, O. M. Bakr, H. N. Alshareef, O. F. Mohammed, *ACS Energy Lett.* **2018**, *3*, 2301.

[6] Z. Zhao, W. Xu, G. Pan, Y. Liu, M. Yang, S. Hua, X. Chen, H. Peng, H. Song, *Mater. Res. Bull.* **2019**, *112*, 142.

[7] C. Bi, S. Wang, W. Wen, J. Yuan, G. Cao, J. Tian, *J. Phys. Chem. C* **2018**, *122*, 5151.

[8] T. Ahmed, S. Seth, A. Samanta, *Chem. Mater.* **2018**, *30*, 3633.

[9] M. Imran, V. Caligiuri, M. Wang, L. Goldoni, M. Prato, R. Krahne, L. De Trizio, L. Manna, *J. Am. Chem. Soc.* **2018**, *140*, 2656.

[10] S. Wang, Y. Wang, Y. Zhang, X. Zhang, X. Shen, X. Zhuang, P. Lu, W. W. Yu, S. V. Kershaw, A. L. Rogach, *J. Phys. Chem. Lett.* **2019**, *10*, 90.

[11] Y. Zhai, X. Bai, G. Pan, J. Zhu, H. Shao, B. Dong, L. Xu, H. Song, *Nanoscale* **2019**, *11*, 2484.

[12] Y. C. Chen, H. L. Chou, J. C. Lin, Y. C. Lee, C. W. Pao, J. L. Chen, C. C. Chang, R. Y. Chi, T. R. Kuo, C. W. Lu, D. Y. Wang, *J. Phys. Chem. C* **2019**, *123*, 2353.

[13] G. Pan, X. Bai, W. Xu, X. Chen, Y. Zhai, J. Zhu, H. Shao, N. Ding, L. Xu, B. Dong, Y. Mao, H. Song, *ACS Appl. Mater. Interfaces* **2020**, *12*, 14195.

[14] S. Zhang, H. Liu, X. Li, S. Wang, *Nano Energy* **2020**, *77*, 105302.

[15] C. Otero-Martínez, D. García-Lojo, I. Pastoriza-Santos, J. Pérez-Juste, L. Polavarapu, *Angew. Chemie - Int. Ed.* **2021**, *60*, 26677.

[16] J. Song, J. Li, X. Li, L. Xu, Y. Dong, H. Zeng, *Adv. Mater.* **2015**, *27*, 7162.

[17] G. Li, F. W. R. Rivarola, N. J. L. K. Davis, S. Bai, T. C. Jellicoe, F. De La Peña, S. Hou, C. Ducati, F. Gao, R. H. Friend, N. C. Greenham, Z. K. Tan, *Adv. Mater.* **2016**, *28*, 3528.

[18] E. P. Yao, Z. Yang, L. Meng, P. Sun, S. Dong, Y. Yang, Y. Yang, *Adv. Mater.* **2017**, *29*, 1606859.

[19] M. K. Gangishetty, S. Hou, Q. Quan, D. N. Congreve, *Adv. Mater.* **2018**, *30*, 1706226.

[20] S. Hou, M. K. Gangishetty, Q. Quan, D. N. Congreve, *Joule* **2018**, *2*, 2421.

[21] S. T. Ochsenbein, F. Krieg, Y. Shynkarenko, G. Rainò, M. V. Kovalenko, *ACS Appl. Mater. Interfaces* **2019**, *11*, 21655.

[22] B. Bin Zhang, S. Yuan, J. P. Ma, Y. Zhou, J. Hou, X. Chen, W. Zheng, H. Shen, X. C. Wang, B. Sun, O. M. Bakr, L. S. Liao, H. T. Sun, *J. Am. Chem. Soc.* **2019**, *141*, 15423.

[23] Y. Shynkarenko, M. I. Bodnarchuk, C. Bernasconi, Y. Berezovska, V. Verteletskyi, S. T. Ochsenbein, M. V. Kovalenko, *ACS Energy Lett.* **2019**, *4*, 2703.

[24] F. Meng, X. Liu, X. Cai, Z. Gong, B. Li, W. Xie, M. Li, D. Chen, H. L. Yip, S. J. Su, *Nanoscale* **2019**, *11*, 1295.

[25] Y. S. Shin, Y. J. Yoon, K. T. Lee, J. Jeong, S. Y. Park, G. H. Kim, J. Y. Kim, *ACS Appl.*

Mater. Interfaces **2019**, *11*, 23401.

[26] H. Wang, X. Zhao, B. Zhang, Z. Xie, *J. Mater. Chem. C* **2019**, *7*, 5596.

[27] F. Ye, H. Zhang, P. Wang, J. Cai, L. Wang, D. Liu, T. Wang, *Chem. Mater.* **2020**, *32*, 3211.

[28] T. Chiba, J. Sato, S. Ishikawa, Y. Takahashi, H. Ebe, S. Sumikoshi, S. Ohisa, J. Kido, *ACS Appl. Mater. Interfaces* **2020**, *12*, 53891.

[29] F. Yang, H. Chen, R. Zhang, X. Liu, W. Zhang, J. Zhang, F. Gao, L. Wang, *Adv. Funct. Mater.* **2020**, *30*, 1908760.

[30] F. Chen, L. Xu, Y. Li, T. Fang, T. Wang, M. Salerno, M. Prato, J. Song, *J. Mater. Chem. C* **2020**, *8*, 13445.

[31] T. Chiba, S. Ishikawa, J. Sato, Y. Takahashi, H. Ebe, S. Ohisa, J. Kido, *Adv. Opt. Mater.* **2020**, *8*, 2000289.

[32] H. Shao, Y. Zhai, X. Wu, W. Xu, L. Xu, B. Dong, X. Bai, H. Cui, H. Song, *Nanoscale* **2020**, *12*, 11728.

[33] Y. R. Park, H. H. Kim, S. Eom, W. K. Choi, H. Choi, B. R. Lee, Y. Kang, *J. Mater. Chem. C* **2021**, *9*, 3608.

[34] X. Wang, T. Bai, X. Meng, S. Ji, R. Zhang, D. Zheng, B. Yang, J. Jiang, K. L. Han, F. Liu, *ACS Appl. Mater. Interfaces* **2022**, *14*, 46857.

[35] F. Zhang, H. Zhong, C. Chen, X. G. Wu, X. Hu, H. Huang, J. Han, B. Zou, Y. Dong, *ACS Nano* **2015**, *9*, 4533.

[36] M. B. Teunis, K. N. Lawrence, P. Dutta, A. P. Siegel, R. Sardar, *Nanoscale* **2016**, *8*, 17433.

[37] S. Sun, D. Yuan, Y. Xu, A. Wang, Z. Deng, *ACS Nano* **2016**, *10*, 3648.

[38] Z. Liang, S. Zhao, Z. Xu, B. Qiao, P. Song, D. Gao, X. Xu, *ACS Appl. Mater. Interfaces* **2016**, *8*, 28824.

[39] G. Li, H. Wang, T. Zhang, L. Mi, Y. Zhang, Z. Zhang, W. Zhang, Y. Jiang, *Adv. Funct. Mater.* **2016**, *26*, 8478.

[40] S. Seth, A. Samanta, *Sci. Rep.* **2016**, *6*, 37693.

[41] J. A. Sichert, Y. Tong, N. Mutz, M. Vollmer, S. Fischer, K. Z. Milowska, R. García Cortadella, B. Nickel, C. Cardenas-Daw, J. K. Stolarczyk, A. S. Urban, J. Feldmann, *Nano Lett.* **2015**, *15*, 6521.

[42] Y. Bekenstein, B. A. Koscher, S. W. Eaton, P. Yang, A. P. Alivisatos, *J. Am. Chem. Soc.* **2015**, *137*, 16008.

[43] Q. A. Akkerman, S. G. Motti, A. R. Srimath Kandada, E. Mosconi, V. D'Innocenzo, G. Bertoni, S. Marras, B. A. Kamino, L. Miranda, F. De Angelis, A. Petrozza, M. Prato, L. Manna, *J. Am. Chem. Soc.* **2016**, *138*, 1010.

[44] I. Levchuk, P. Herre, M. Brandl, A. Osvet, R. Hock, W. Peukert, P. Schweizer, E. Spiecker, M. Batentschuk, C. J. Brabec, *Chem. Commun.* **2017**, *53*, 244.

[45] B. J. Bohn, Y. Tong, M. Gramlich, M. L. Lai, M. Döblinger, K. Wang, R. L. Z. Hoye, P. Müller-Buschbaum, S. D. Stranks, A. S. Urban, L. Polavarapu, J. Feldmann, *Nano Lett.* **2018**, *18*, 5231.

[46] Z. Yuan, Y. Shu, Y. Xin, B. Ma, *Chem. Commun.* **2016**, *52*, 3887.

[47] J. Cho, Y. H. Choi, T. E. O'Loughlin, L. De Jesus, S. Banerjee, *Chem. Mater.* **2016**, *28*, 6909.

[48] J. Shamsi, P. Rastogi, V. Caligiuri, A. L. Abdelhady, D. Spirito, L. Manna, R. Krahne, *ACS Nano* **2017**, *11*, 10206.

[49] G. H. Ahmed, J. Yin, R. Bose, L. Sinatra, E. Alarousu, E. Yengel, N. M. Alyami, M. I. Saidaminov, Y. Zhang, M. N. Hedhili, O. M. Bakr, J. L. Brédas, O. F. Mohammed, *Chem. Mater.* **2017**, *29*, 4393.

[50] J. Shamsi, D. Kubicki, D. Kubicki, M. Anaya, Y. Liu, K. Ji, K. Frohna, C. P. Grey, R. H. Friend, S. D. Stranks, *ACS Energy Lett.* **2020**, *5*, 1900.

[51] S. Peng, Q. Wei, B. Wang, Z. Zhang, H. Yang, G. Pang, K. Wang, G. Xing, X. W. Sun, Z. Tang, *Angew. Chemie - Int. Ed.* **2020**, *59*, 22156.

[52] D. Rossi, X. Liu, Y. Lee, M. Khurana, J. Puthenpurayil, K. Kim, A. V. Akimov, J. Cheon, D. H. Son, *Nano Lett.* **2020**, *20*, 7321.

[53] S. Kumar, J. Jagielski, S. Yakunin, P. Rice, Y. C. Chiu, M. Wang, G. Nedelcu, Y. Kim, S. Lin, E. J. G. Santos, M. V. Kovalenko, C. J. Shih, *ACS Nano* **2016**, *10*, 9720.

[54] D. Yang, Y. Zou, P. Li, Q. Liu, L. Wu, H. Hu, Y. Xu, B. Sun, Q. Zhang, S. T. Lee, *Nano Energy* **2018**, *47*, 235.

[55] C. Zhang, Q. Wan, B. Wang, W. Zheng, M. Liu, Q. Zhang, L. Kong, L. Li, *J. Phys. Chem. C* **2019**, *123*, 26161.

[56] W. Deng, X. Jin, Y. Lv, X. Zhang, X. Zhang, J. Jie, *Adv. Funct. Mater.* **2019**, *29*, 1903861.

[57] R. L. Z. Hoye, M. L. Lai, M. Anaya, Y. Tong, K. Gałkowski, T. Doherty, W. Li, T. N. Huq, S. Mackowski, L. Polavarapu, J. Feldmann, J. L. Macmanus-Driscoll, R. H. Friend, A. S. Urban, S. D. Stranks, *ACS Energy Lett.* **2019**, *4*, 1181.

[58] S. Peng, Z. Wen, T. Ye, X. Xiao, K. Wang, J. Xia, J. Sun, T. Zhang, G. Mei, H. Liu, B. Xu, X. Li, R. Chen, G. Xing, K. Wang, Z. Tang, *ACS Appl. Mater. Interfaces* **2020**, *12*, 31863.

[59] W. Yin, M. Li, W. Dong, Z. Luo, Y. Li, J. Qian, J. Zhang, W. Zhang, Y. Zhang, S. V. Kershaw, X. Zhang, W. Zheng, A. L. Rogach, *ACS Energy Lett.* **2021**, *6*, 477.
